# Assessment of SAPT(DFT) with meta-GGA functionals

**DOI:** 10.1007/s00894-020-4340-9

**Published:** 2020-04-15

**Authors:** Michał Hapka, Marcin Modrzejewski, Grzegorz Chałasiński, Małgorzata M. Szczęśniak

**Affiliations:** 1 Department of Chemistry, University of Michigan, Ann Arbor, MI 48109 USA; 2 Faculty of Chemistry, University of Warsaw, Pasteura 1, 02-093 Warszawa, Poland; 3 Department of Chemistry, Oakland University, Rochester, MI 48309 USA

**Keywords:** Symmetry-adapted perturbation theory, SAPT(DFT), Intermolecular interactions, Meta-GGA, Dispersion energy, Electrostatic energy, Exchange repulsion, DFT-SAPT

## Abstract

This work examines the suitability of meta-GGA functionals for symmetry-adapted perturbation theory (SAPT) calculations. The assessment is based on the term-by-term comparison with the benchmark SAPT variant based on coupled-cluster singles and doubles description of monomers, SAPT(CCSD). Testing systems include molecular complexes ranging from strong to weak and the He dimer. The following nonempirical meta-GGAs are examined: TPSS, revTPSS, MVS, SCAN, and SCAN0 with and without the asymptotic correction (AC) of the exchange-correlation potential. One range-separated meta-GGA functional, LC-PBETPSS, is also included. The AC-corrected pure meta-GGAs (with the exception of MVS) represent a definite progress in SAPT(DFT) compared to pure GGA, such as PBEAC, with their more consistent predictions of energy components. However, none of the meta-GGAs is better than the hybrid GGA approach SAPT(PBE0AC). The SAPT(DFT) electrostatic energy offers the most sensitive probe of the quality of the underlying DFT density. Both SCAN- and TPSS-based electrostatic energies agree with reference to within 5% or better which is an excellent result. We find that SCAN0 can be used in SAPT without the AC correction. The long-range corrected LC-PBETPSS is a reliable performer both for the components and total interaction energies.

**Electronic supplementary material:**

The online version of this article (10.1007/s00894-020-4340-9) contains supplementary material, which is available to authorized users.

## Introduction

Symmetry-adapted perturbation theory (SAPT) is an effective means of computing the interaction energies of non-covalent interactions with direct insights into their composition [1]. Non-covalent interactions result from a delicate balance of the attractive and repulsive components some of which, e.g., the dispersion energy, have no classical correspondent. The repulsive contributions are equally important and, with the exception of electrostatics, also nonclassical in nature because they originate from the antisymmetry requirement [2]. The only rigorous way to obtain the dispersion interactions, as well as the exchange contributions (in a weak symmetry forcing scheme), is via the application of SAPT methodology [3]. In its first many-electron formulation SAPT (the so called many-body, MB-SAPT) was a double perturbation theory expansion with respect to two perturbations: intermolecular interaction operator and the intramonomer correlation operator [1, 4]. In this theory, many terms are needed to correctly describe particular interaction energy term. For example, the exact description of the electrostatic energy requires an infinite expansion in terms of intramonomer correlation [5, 6]. By contrast, if the monomers were already correlated, the electrostatic effect, as well as any other contribution, would be more straightforward to compute and interpret.

Advantages due to bypassing the double perturbation expansion have been explored in notable recent SAPT extensions. Holzer and Klopper developed SAPT based on quasiparticle energies and response functions from the GW method [7]. Boese and Jansen used accurate densities to construct Kohn-Sham (KS) exchange-correlation potentials for use in SAPT [8]. Korona employed CCSD density matrices and response functions in the formulation of SAPT(CCSD) which also bypasses double-perturbation theory [9]. Finally, Hapka et al. investigated the extent to which the complete active space monomers’ wave functions recover the intrasystem correlation effects on the dispersion energy [10, 11].

If the density functional theory (DFT) were to be used for describing the monomer wave functions, the intrasystem correlation effects can be captured (at least in principle) giving rise to the simplest way of bypassing double perturbation SAPT expansion. This idea was independently developed by the groups of Szalewicz [5, 12–14] and Jansen [15–18] and known as SAPT(DFT) (or DFT-SAPT). The promise of this theory lies in the fact that if we had the accurate density functional and its derivatives, the theory would be exact to the second-order as far as the polarization terms are concerned. The exchange terms require one- and two-particle density matrices which cannot in principle be reproduced even with the exact KS determinant. In practice, the asymptotic form of the first-order exchange should be accurate in SAPT(DFT) [14]. Furthermore, the numerical evidence shows the excellent agreement between first- and second-order exchange terms and the available benchmarks [9, 14].

A path toward further improvement of SAPT(DFT) is to step-up onto a higher rung of the Jacob’s ladder [19], i.e., to seek DFT functionals that are closer to the ideal of the accurate functional. To date, mainly GGA functionals and their hybrids have been employed in SAPT. As far as meta-GGAs are concerned, only Minnesota M05 [20] and M06 [21] functionals and their hybrids were examined in Ref. [9] with mixed results. Namely, M05 functional yielded decent electrostatic and dispersion components whereas in M06 these terms were exceptionally poor. The heavily parametrized nature of these functionals makes it difficult to ascertain what was the reason behind this disparity.

In the present paper, we intend to systematically examine a newer group of nonempirical meta-GGAs for their suitability in SAPT(DFT). Meta-GGA exchange-correlation energies depend on kinetic energy density, τ, in addition to density and its gradient as GGAs do. This gives them more functional flexibility to satisfy a larger number of exact constraints. For example, at the GGA level, it is impossible to detect one-electron densities, e.g., hydrogen atom density, which leads to the one-electron self-interaction error. It is only the orbital-dependent τ which offers this capability. Nonempirical meta-GGA functionals are constructed to satisfy the constraints that the exact functional is known to obey. The latest fruit of this effort led by the Perdew, Ruzsinszky, and Sun team is the functional SCAN (strongly constrained appropriately normed) which satisfies all 17 known exact constraints (among which there are, for example, tighter lower bound for exchange, gradient expansion accurate to 4th order, one-electron self-correlation equal zero, etc.) [22]. Newer meta-GGAs, such as MVS [23] and SCAN, reputably distinguish between paradigm bonding cases in chemistry and physics: covalent, metallic, and noncovalent. This is because of the skillful incorporation of kinetic energy density in the form of the nondimensional kinetic ingredient: α = (τ - τ^W^)/ τ^UEG^ where τ^W^ is von Weizsacker kinetic energy density of one-electron system and τ^UEG^ stands for the kinetic energy density of the uniform electron gas. α differs for covalent, metallic, and noncovalent bonds. It is interesting if this ability reflects on density functional’s performance in SAPT. Another way of incorporating meta-GGA ingredients is through the range-separation procedure build on the Becke-Roussel exchange hole [24] that includes both τ and the Laplacian of density. One such range-separated meta-GGAs is tested here in SAPT as well. The results for meta-GGAs will be compared with the current SAPT(DFT) “standard-bearer” GGAs, PBEAC, and PBE0AC, gauging the results against SAPT(CCSD) benchmarks [9]. To complete the comparison, we also employ the wave function SAPT; the details of which will be described in the next section.

## Computational details

The framework for evaluation of new functionals’ performance is a term-by-term comparison with SAPT(CCSD) of Korona [9] used as reference. The test set includes the same dimers as in Ref. [9]. The set of molecules ranges in strength of interactions from very strong hydrogen bonds (FHF)^−^, where proton is shared, to very weak van der Waals complexes involving He bound to a molecule, e.g., CO_2_. It also includes third period elements. Following Ref. [9] two subsets are considered. Larger set S_1_ containing 21 dimers was tested in the smaller aug-cc-pVDZ basis set [25, 26]. The S_2_ set is its subset from which six large dimers were removed. The S_2_ set was tested in the larger, aug-cc-pVTZ, basis set. This choice is dictated by the high computational demands of SAPT(CCSD) reference calculations. Compared to Ref. [9], we also computed methane dimer SAPT(CCSD) benchmark data in aug-cc-pVTZ basis set and included them in the set S_2_. Both sets are referred to as TK21. Another important paradigm is He_2_. Here, we conduct a similar term-by-term comparison as in the work of Cencek and Szalewicz [27, 28] thus extending their insights to meta-GGAs: pure, hybrid, and range-separated hybrid. The details including the basis set, geometry, and the reference values are the same as in their work.

The following functionals are included in the present study: TPSS [29], revTPSS [30], MVS [23], SCAN [22], and SCAN0 [31] representing a progression in the meta-GGA design. Range-separated meta-GGAs are still quite rare. We include one such functional, LC-PBETPSS, designed using the range-separation method proposed in Ref. [32]. In this method, the short-range exchange is obtained using Becke-Roussel exchange hole which is exact for hydrogenic systems and contains in its formulation the meta-GGA ingredients. If combined with PBE exchange, this method elevates PBE to the meta rung. Such a meta-GGA exchange is then combined with the TPSS correlation thus forming the LC-PBETPSS meta-GGA functional. If range-separated functionals are to be used in SAPT, the range-separation parameters should be optimized so as to minimize the difference, Δ_XC_, between the vertical ionization potential, *IP*, and the negative energy of the highest occupied molecular orbital (HOMO), e_HOMO_, the method known as the IP tuning. In this work, we used the monomer range-separation parameters previously optimized for LC-ωPBE (i.e., for range-separated GGA) from Ref. [33].

Range-separated hybrids employ v_XC_ potentials which converge to the correct − 1/r asymptote [34]. This is not the case for the other meta-GGAs and their hybrids. To remedy this problem, v_XC_ potentials are equipped with the so called asymptotic correction (AC) which ensures that the correct v_XC_ → − 1/r + Δ_XC_ limit is reached. In the present work, the gradient-regulated asymptotic correction (GRAC) [35] is employed and applied to TPSS, revTPSS, MVS, SCAN, and SCAN0 functionals. To accommodate the asymptotic correction to meta-GGAs, we have supplied the existing GGA implementation with the kinetic energy derivatives scaled by (1-f^GRAC^), where f^GRAC^ is the GRAC interpolation factor [35, 36]. For practical reasons, we have omitted the extra terms described in Refs. [27, 36]. The monomer ionization potentials needed for the correction are taken from experiment for consistency with results of Ref. [9].

The SAPT(DFT) theory represents the interaction energy *E*_int_ as

1 $$ {E}_{\mathrm{int}}={E}_{\mathrm{elst}}^{(1)}+{E}_{\mathrm{exch}}^{(1)}+{E}_{\mathrm{ind}}^{(2)}+{E}_{\mathrm{exch}-\mathrm{ind}}^{(2)}+{E}_{\mathrm{disp}}^{(2)}+{E}_{\mathrm{exch}-\mathrm{disp}}^{(2)}. $$

All exchange energies are evaluated in the S^2^ approximation (S refers to the overlap integral) to permit the direct comparison with the SAPT(CCSD) benchmarks using this approximation. The second-order terms ${E}_{\mathrm{ind}}^{(2)}$ and ${E}_{\mathrm{disp}}^{(2)}$, and their respective exchange counterparts, are obtained from the coupled Kohn-Sham (KS) density-response functions with the underlying pure ALDA (adiabatic local density) approximation or hybrid kernels, depending on the type of functional. All calculations were performed with the internally modified Molpro 2012 program suite [37] with no density fitting.

The MVS, SCAN, and SCAN0 computations involved an extra dense Log3 radial grid (*n*_*r*_ = 250) (from Ref. [38]).

The wave function SAPT (MB-SAPT) is also included in the comparison. The MB-SAPT version, denoted SAPT2+(CCD), includes the following elements:

2 $$ {E}_{\mathrm{int}}^{\mathrm{SAPT}2+\left(\mathrm{CCD}\right)}={E}_{\mathrm{elst}}^{(10)}+{E}_{\mathrm{elst},\mathrm{resp}}^{(12)}+{E}_{\mathrm{exch}}^{(10)}+{E}_{\mathrm{exch}}^{(11)}+{E}_{\mathrm{exch}}^{(12)}+{E}_{\mathrm{ind},\mathrm{resp}}^{(20)}+{}^t{E}_{\mathrm{ind}}^{(22)}+{E}_{\mathrm{exch}-\mathrm{ind},\mathrm{resp}}^{(20)}+{}^t{E}_{\mathrm{exch}-\mathrm{ind}}^{(22)}+{E}_{\mathrm{disp},\mathrm{CCD}}^{(2)}+{E}_{\mathrm{exch}-\mathrm{disp}}^{(20)}. $$

All the components except for the dispersion energy are the same as in the SAPT2 variant, as described by Hohenstein and Sherrill [39]. The second-order dispersion term ${E}_{\mathrm{disp},\mathrm{CCD}}^{(2)}$ is calculated at the CCD + ST (CCD) level of theory as first proposed by Williams et al. [40]. The total interaction energy excludes the so called δ_HF_ term (see, e.g., [41]). The SAPT2+(CCD) calculations were performed using Psi4 program [42] with the use of natural orbital truncation techniques for the CCD + ST (CCD) dispersion energy [43]. The wave function SAPT denoted SAPT(HF) involves all the terms computed analogously to Eq. (1) except for employing the Hartree-Fock (HF) wave function instead of KS. Note that in SAPT(HF), the exchange-dispersion energy is obtained at the coupled level of theory, whereas in SAPT2 + (CCD), this component is included within the uncoupled approximation.

The relative percent errors in energies (E_i_) with respect to reference values (E_i,ref_) shown in the plots and in the tables below are defined as:

$$ \Delta \left(\%\right)=\left({\mathrm{E}}_{\mathrm{i}}-{\mathrm{E}}_{\mathrm{i},\mathrm{ref}}\right)/\mid {\mathrm{E}}_{\mathrm{i},\mathrm{ref}}\mid \times 100. $$

## Results and discussion

- He_2_ dimer

The analysis begins with the interaction energy components of the He dimer shown in Table 1. The previous SAPT(DFT) suitability study of this type by Cencek and Szalewicz [27] involved only GGAs and their hybrids. It is here extended to include SCAN, SCAN0 with AC-corrected variants, as well as LC-PBETPSS, a range separated meta-GGA. As seen in Table 1, SAPT(DFT) based on SCAN represents a dramatic improvement with respect to PBE. The errors in the first-order terms are reduced by one half, whereas those in the induction and exchange-induction are reduced by nearly an order of magnitude. The dispersion energy in SCAN is only 4% away from the reference. When compared with PBE0, the SCAN components are considerably closer to the reference values. This observation goes against the conventional wisdom which assumes that SCAN is comparable in performance to hybrid GGAs. Hybrid SCAN0 affords further improvements in energy components particularly for dispersion energy. Adding the AC correction brings the expected improvement to both GGA and meta-GGAs’ results. Although individual SCANAC and SCAN0AC components exhibit larger errors compared to PBE0AC, the total interaction energies are in better agreement with the Gaussian geminal reference. This results from the error compensation due to systematic underestimation of all the energy contributions.

**Table 1:** Percent errors in He_2_ with respect to the reference^a^ for GGA and meta-GGA functionals in SAPT (DFT) at equilibrium separation of 5.6 bohr. All calculations were performed in d-aug-cc-pV5Z + mid-bond functions (the same as in Ref. [28]). Units are cm^−1^

Method	${E}_{\mathrm{elst}}^{(1)}$	${E}_{\mathrm{exch}}^{(1)}$	${E}_{\mathrm{ind}}^{(2)}$	${E}_{\mathrm{exch}-\mathrm{ind}}^{(2)}$	${E}_{\mathrm{disp}}^{(2)}$	${E}_{\mathrm{exch}-\mathrm{disp}}^{(2)}$	*E*_*int*_
Reference values^a^	− 1.187	8.54	− 0.196	0.177	− 15.565	0.515	− 7.716
PBE	− 101.7	118.0	− 156.5	165.9	− 26.0	85.4	68.0
SCAN	− 48.4	60.3	− 19.2	20.9	− 4.4	18.5	51.7
PBE0	− 55.9	65.2	− 83.5	83.0	− 14.2	46.5	37.8
SCAN0	− 27.6	35.1	− 15.6	14.2	− 1.4	10.1	32.3
PBE0AC	0.5	− 2.9	5.4	− 14.3	− 3.2	3.5	− 9.5
SCANAC	9.2	− 11.9	15.9	− 24.4	3.8	− 8.3	4.6
SCAN0AC	8.4	− 10.0	13.3	− 21.6	3.9	− 8.1	− 2.5
LC-PBETPSS(1.02)^b^	2.9	− 3.0	4.7	− 12.9	3.6	− 5.3	3.8

^a)^Reference values are from Gaussian type geminal calculations as quoted in Ref. [28]

^b)^The value of IP optimized range-separation parameter shown in parenthesis

The range-separated meta-GGA LC-PBETPSS yields both the components and the total interaction energy in close agreement with the reference values. This signifies that the density, density matrix, and response properties (static and frequency-dependent) are correctly described in this functional. Its resulting error of 3.8% in total interaction energy compares favorably with the previously studied range-separated GGA in Ref. [28] that predicted 5.3% error in the same basis set.

We conclude that in two-electron systems, and for representative functionals PBE and SCAN, adding meta-ingredients to GGA is more effective than the hybridization of GGA, in improving the energy components. The quality of dispersion energy in SCAN and SCAN0 in two-electron systems is worth noting. The electrostatic energy improves only upon inclusion of the AC correction. Below, we examine how these conclusions hold-up in SAPT (DFT) for polyelectron monomers.

b. Overall suitability for variety of interaction types

In Table 2, we show the interaction energies of all the complexes in TK21 S_1_ set and the percentage errors of SAPT (DFT) with respect to SAPT (CCSD) for all considered meta-GGAs. The complexes in TK21 set can be grouped into ion-molecule interactions, hydrogen-bonded systems from typical (H_2_O dimer) to unorthodox (C_2_H_6_-HCN, i.e., proton donating to a methyl group), a hydrocarbon dimer (CH_4_ dimer), interactions of quadrupolar molecules from weak (N_2_ dimer) to donor-acceptor (PCCP dimer), to typical van der Waals systems (molecule-He and Ar_2_). The objective here is to find if SAPT(DFT) based on meta-GGAs succeeds for any particular type of complexes.

**Table 2:** Reference SAPT(CCSD) interaction energies (in mH) for TK21 molecule set and the percentage errors in SAPT(DFT) using the listed meta-GGA functionals. PBE0AC is included for comparison. Basis set is aug-cc-pVDZ

Complex	E_int_ SAPT (CCSD)	SAPT (DFT) % errors											
		PBE0 AC	TPSS	TPSSAC	revTPSS	revTPSS AC	MVS	MVSAC	SCAN	SCANAC	SCAN0	SCAN0 AC	LC-PBETPSS
F^−^-HF	− 58.98	− 6.5	6.9	8.8	9.9	11.7	− 4.9	− 4.3	3.8	5.0	− 11.5	− 11.2	− 11.5
F^−^-H_2_O	− 25.59	− 0.5	12.7	14.2	15.0	16.4	− 1.8	− 1.3	7.8	8.7	− 6.8	− 6.5	1.1
Na^+^-H_2_O	− 36.19	− 0.8	3.7	2.1	4.3	2.7	− 1.4	− 2.0	0.8	− 0.5	− 2.6	− 3.1	− 3.6
HF-HF	− 4.78	− 4.4	23.4	8.2	24.4	9.5	− 2.5	− 5.5	7.7	0.6	− 9.2	− 11.3	− 18.8
H_2_O-H_2_O	− 5.26	− 1.8	23.8	12.9	25.0	14.4	− 5.9	− 5.9	9.8	6.2	− 7.4	− 7.3	− 10.6
NH_3_-CH_4_	− 0.91	16.4	26.6	23.8	28.1	26.4	3.6	16.5	14.0	21.7	4.0	18.0	11.9
NH_3_-H_2_O	− 6.92	3.4	18.5	14.8	19.4	16.0	− 5.1	− 1.0	10.6	12.0	− 3.5	− 0.1	− 5.4
C_2_H_6_-HCN	− 1.22	3.0	21.4	16.0	24.2	19.8	− 1.0	8.3	9.8	13.4	− 3.6	3.7	− 1.5
CH_4_-CH_4_	− 0.72	24.3	22.0	24.3	21.3	24.5	16.3	29.7	17.9	28.1	11.4	26.9	14.1
C_2_H_2_-C_2_H_2_-PD	− 1.72	− 5.2	17.1	4.7	15.6	3.8	− 18.2	− 13.7	1.6	− 0.7	− 10.5	− 10.6	1.4
C_2_H_2_-C_2_H_2_-S	− 0.21	26.4	55.7	16.3	56.5	18.0	77.2	71.4	58.5	38.0	55.7	44.0	64.4
C_2_H_2_-C_2_H_2_-T	− 1.74	− 9.8	15.2	1.1	14.3	1.0	− 19.8	− 13.9	−1.7	− 4.4	− 14.2	− 14.3	− 1.8
NCCN-NCCN	− 2.73	13.8	21.1	16.1	21.1	16.4	11.3	17.0	13.8	15.3	8.1	10.9	7.0
PCCP-PCCP	− 3.18	16.9	25.1	22.8	24.3	22.5	14.6	25.0	21.7	26.8	16.4	20.3	32.5
N_2_-N_2_	− 0.38	11.3	28.0	2.9	26.8	2.9	15.3	8.3	21.8	9.8	17.0	15.4	16.3
P_2_-P_2_	− 1.01	11.9	19.3	10.2	18.5	10.3	8.0	17.5	16.8	18.9	15.4	18.1	32.5
N_2_O-He (GM)	− 0.16	1.3	57.9	11.3	54.4	11.1	38.5	16.7	47.1	14.9	25.1	8.3	− 2.0
N_2_O-He (LM)	− 0.09	6.7	65.0	16.3	60.0	15.1	44.3	23.7	48.5	17.6	25.0	9.5	13.8
CO_2_-He (GM)	− 0.10	2.9	93.1	13.3	87.1	12.7	58.3	19.1	71.4	17.1	34.1	7.5	− 7.5
CO_2_-He (LM) Ar-Ar	− 0.07 − 0.20	4.3 − 2.5	62.6 24.1	7.6 − 3.5	58.7 23.6	7.2 − 3.5	39.8 7.8	17.0 2.7	45.6 15.3	10.6 1.6	21.2 7.0	5.6 0.5	11.0 5.6
Mean % error		5.3	30.6	11.6	30.1	12.3	13.1	10.7	21.1	12.4	8.2	5.9	7.1
MAE		8.3	30.6	12.3	57.8	12.6	18.8	15.3	21.0	12.9	14.8	12.1	13.0

An analog of Table 2 for the S_2_ set of dimers computed in the aug-cc-pVTZ basis set is included in the Supplementary Information as Table S1.

In ion-molecule interactions, MVS and SCAN perform very well. In this group, the addition of asymptotic correction appears to be of no help. In hydrogen-bonded complexes, MVS and SCAN are also good performers. AC correction is marginally helpful; the addition of HF-exchange to SCAN is beneficial. Although the range separated HF-exchange in LC-PBETPSS appears ineffective even in H_2_O and HF dimers, its effectiveness improves greatly in the larger basis set (see Table S1). It is noteworthy that TPSS and revTPSS perform poorly in this group and the addition of AC still keeps the errors in the double-digit range.

For weakly interacting CH_4_ dimer, a simplest hydrocarbon interaction, all the functionals except SCAN0 and LC-PBETPSS work poorly. Since SAPT(HF) [9] performs very well for this system, we hypothesize that the problem lies in the quality of DFT exchange at low reduced gradients.

In the acetylene dimer, two structures PD and T are very well reproduced by SCAN and LC-PBETPSS as well as all the AC-corrected SCAN. The S structure is very weakly bound; consequently, even small inaccuracies lead to large double-digit errors. The case of (NCCN)_2_ and (PCCP)_2_ is interesting: Both are donor-acceptor complexes for which SAPT generally works poorly [44]. Nevertheless, for the former, SAPT based on LC-PBETPSS and SCAN0 performs well. The remaining complexes are typical van der Waals systems dominated by dispersion in subtle balance with the repulsive exchange. Again, the errors appear large because the effects are very small. Here, both the AC correction and inclusion of exact exchange bring a significant improvement, and SCAN0AC and LC-PBETPSS are the only reliable performers. Ar_2_ is well reproduced by the majority of functionals, particularly those AC-corrected.

c. Performance for electrostatic energy

**Fig. 1:**
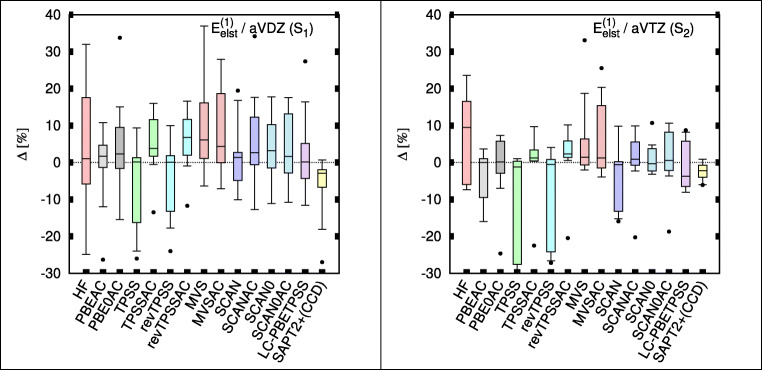
Box plots for electrostatic energies of TK21 set in aug-cc-pVDZ (aVDZ; left) and aug-cc-pVTZ (aVTZ; right). Box includes 50% of error range, and the whiskers include 95% and 90% error range for aVDZ and aVTZ, respectively; the horizontal bars describe the median, and the dots describe the largest outlier. Errors are with respect to SAPT(CCSD) benchmarks [9]

Figure 1 shows relative percent errors from different SAPT(DFT) methods in the form of box-and-whiskers plots; two wave function-based methods SAPT(HF) and SAPT2+(CCD) are added for comparison. In TPSS and revTPSS, the electrostatic energy clearly benefits from the inclusion of the AC correction. These two afford some of the best electrostatic energies overall. By contrast, the changes in electrostatic energies brought by AC in MVS, SCAN, and SCAN0 are unclear. In the aug-cc-pVDZ basis set, those functionals show little or no improvement upon the GRAC correction. In the larger basis (Fig. 1 right panel), only SCAN improves (we emphasize that aug-cc-pVTZ calculations are performed on a smaller subset of complexes S_2_). Note that even without the AC correction SCAN0 is a reliable performer in both basis sets.

The electrostatic energy is a sensitive measure of the quality of density; from this, we can conclude that most of SCF DFT densities considered here are superior to the HF density, against the claims of the latter’s superiority (see, e.g., Ref. [45]).

d. Performance for first-order exchange

**Fig. 2:**
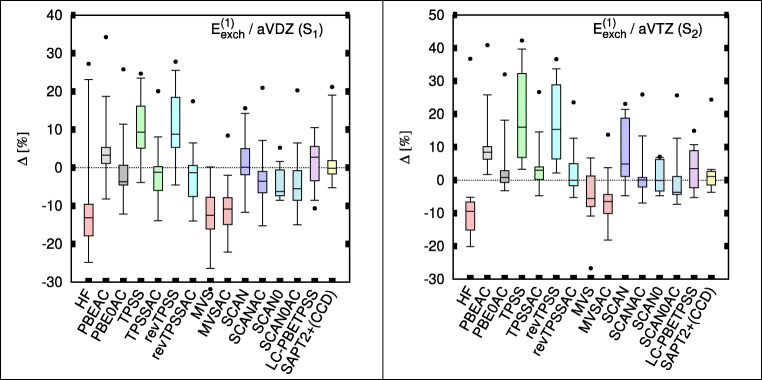
Box plots for first-order exchange of TK21 molecule set in aug-cc-pVDZ (aVDZ; left) and aug-cc-pVTZ (aVTZ; right). Box includes 50% of error range, and the whiskers include 95% and 90% error range for aVDZ and aVTZ, respectively; the horizontal bars describe the median, and the dots describe the largest outlier. Errors are with respect to SAPT(CCSD) benchmarks [9]

As stated earlier, the first-order exchange presents more of a challenge for SAPT(DFT) than the electrostatic term because it requires the accurate description of the entire density matrix in addition to electron density. Figure 2 shows relative errors in the first-order exchange repulsion. All DFT methods are again superior to HF. The AC correction generally benefits TPSS and revTPSS. These two functionals again yield some of the best exchange terms. SCAN0 is an excellent performer even without AC. MVS, similarly, appears little affected by AC and its results are poor. Our LC-meta-GGA performs reasonably well. The larger basis set results confirm the trend with MVSAC as an outlier. The wave function SAPT2 performs very well with the exception of one or two outliers, specifically, the P-containing molecules and ethyne dimer in the S configuration (see also Tables S3 and S10 in the Supplemental Information).

e. Induction and exchange induction

**Fig. 3:**
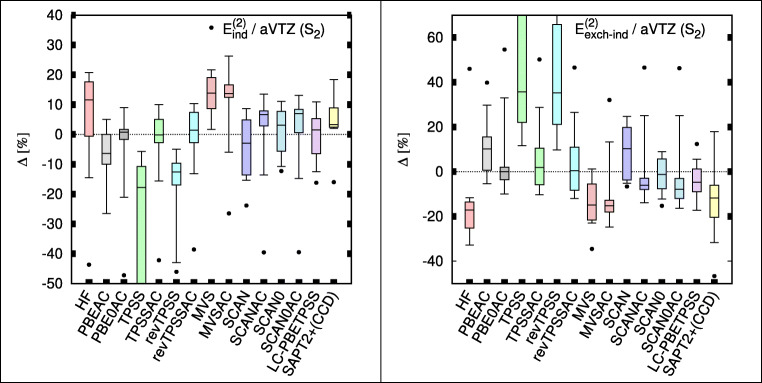
Box plots for the induction (left) and exchange-induction (right) energies of TK21 set in aug-cc-pVTZ (aVTZ) basis set. Box includes 50% of error range and the whiskers include 90% error range; the horizontal bars describe the median, and the dots describe the largest outlier. Errors are with respect to SAPT(CCSD) benchmarks [9]

Figure 3 presents the errors in the induction and exchange-induction terms of TK21 set. To reiterate, the induction and dispersion terms (see below) as well as their exchange counterparts are evaluated using coupled KS response theory. This is important because the corresponding uncoupled terms have one order of magnitude larger errors. The plots indicate particularly poor performance of TPSS and revTPSS and their dramatic improvement upon the AC correction. MVS on the other hand shows no improvement. The remaining meta-GGAs show uniform good performance. The SAPT2+(CCD) results will be discussed below.

f. Dispersion and exchange-dispersion energies

**Fig. 4:**
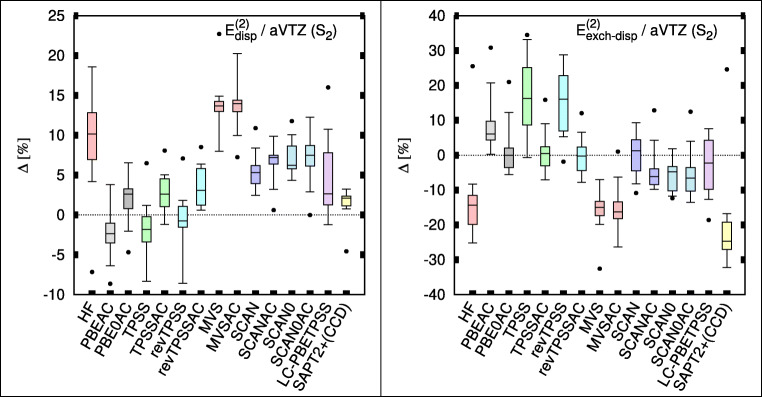
Box plots for the dispersion (left) and exchange-dispersion (right) energies of TK21 set in aug-cc-pVTZ (aVTZ) basis set. Box includes 50% of error range, and the whiskers include 90% error range; the horizontal bars describe the median, and the dots describe the largest outlier. Errors are with respect to SAPT(CCSD) benchmarks [9]

Figure 4 shows the errors in the second-order dispersion and exchange-dispersion terms. The two TPSS-based functionals afford surprisingly accurate dispersion energies. The previously noted SCAN performance in He_2_ holds here as well, although based on some error cancelation between an underestimation of both the dispersion attraction and exchange-dispersion repulsion. For exchange-dispersion, the AC-corrected TPSS and revTPSS are excellent. The LC-meta-GGA performs very well for both terms, although the spread of errors is larger than in other functionals. MVSAC acts as an outlier in both the dispersion and its exchange counterpart.

The performance of wave function SAPT2 calls for an explanation at this point. The SAPT2 version considered here evaluates the exchange-induction at the coupled level of theory and with partial account of the intrasystem correlation [46]. The large percent errors in the exchange-induction term are being dominated by van der Waals dimers where exchange-induction effects are close to zero. The exchange-dispersion, on the other hand, was computed at the uncoupled level of theory and in the zeroth order with respect to the intrasystem correlation operator. This approximation leads to double-digit percentage errors due to a severe underestimation of this repulsive term. Note that in SAPT(HF) calculations, the exchange-dispersion energy is obtained at the coupled level of theory which leads to slightly better agreement with the SAPT(CCSD) benchmark.

g. Total interaction energy

**Fig. 5:**
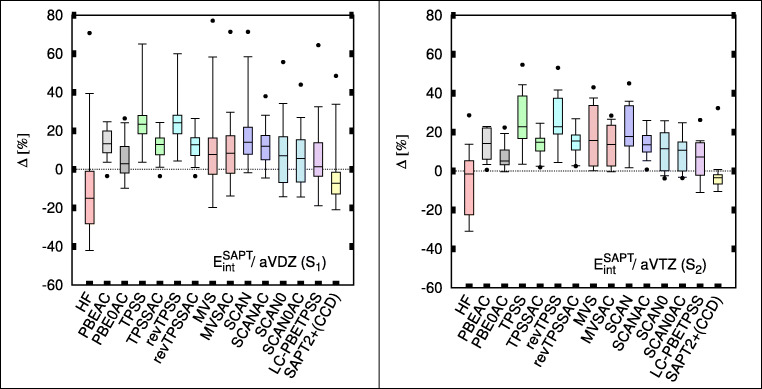
Box plots for the total interaction energy in in aug-cc-pVDZ (aVDZ; left) and aug-cc-pVTZ (aVTZ; right). Box includes 50% of error range, and the whiskers include 95% and 90% error range for aVDZ and aVTZ, respectively; the horizontal bars describe the median, and the dots describe the largest outlier. Errors are with respect to SAPT(CCSD) benchmarks [9]

In Fig. 5 the errors in total interaction energies of TK21 set are displayed. The first question concerns whether the meta-GGAs are clearly better than GGAs in SAPT? GGAs are here represented by PBEAC and PBE0AC, two popular choices of SAPT(DFT) calculations. Three non-hybrid, AC-corrected meta-GGA functionals (TPSS, revTPSS, and SCAN) are better than the non-hybrid GGA – PBEAC. Among the hybrids, SCAN0 and LC-meta-GGA come close to PBE0AC but with a larger spread of errors. In the case of LC-PBETPSS, this is in part due to the fact that the range-separation parameters have not been individually tuned for this functional (see Sec. 2.b). Note that interaction energy errors in MVSAC, which was found above to yield poor individual components, are similar to the SCAN0AC case thus indicating a systematic error cancelation in MVS.

The wave function SAPT2+(CCD) is better than any SAPT(DFT) methods, but outliers do exist. It is clear that for the most part, SAPT based on DFT is always superior to that based on HF.

**Fig. 6:**
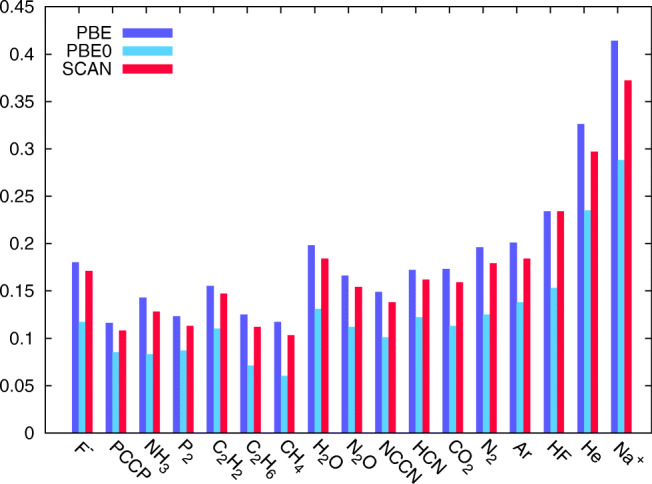
Potential shift Δ_XC_ = IP + e_HOMO_ (in Hartree) for monomers of the TK21 set from aVDZ computation

To shed more light on the above performance, we explore how well meta-GGAs satisfy the Koopmans’ theorem, the issue critical to the SAPT results [15]. Figure 6 shows the comparison of Δ_XC_, that is, the energy value by which the potential needs to be shifted by the AC correction, as computed for TK21 monomers in GGA and meta-GGA and their hybrids. The shown values are with respect to the experimental ionization potentials. Figure 6 demonstrates slight improvements in the theorem’s satisfaction between PBE and SCAN and minimal improvement between PBE0 and SCAN0. Much larger changes result from the addition of HF-exchange to both. The data clearly show that even at the most sophisticated hybrid meta-GGA level, the AC correction is still needed to affix the HOMO energy at the correct value.

The AC correction has some negatives. Namely, the v_XC_ potential so corrected becomes “stray,” i.e., not a functional derivative of any energy formula [47]. For these reasons, one may be interested in finding functionals suitable for SAPT (DFT) without the AC correction. Obviously, LC-corrected functionals serve this purpose. Of the other functionals, SCAN0, while not perfect, could also be useful in SAPT without correcting.

## Summary and conclusions

Meta-GGAs have more sophisticated mathematical forms and hence can satisfy more exact constraints. To what extent satisfying these constraints helps in SAPT(DFT) was our question in this paper. In fact, the constraints apply to the energy formula and their fulfillment is no guarantee that the functional derivative, v_XC_, would similarly improve. In SAPT, it is the v_XC_ potential which is of primary importance. That is why testing of these new functionals within SAPT is needed.

The correct potential asymptote can be secured in two ways: either by using an asymptotic correction, for example, of the GRAC type, or by employing a long-range correction via the range separation of electron-electron interactions. Examining the compatibility of these AC and LC corrections with the new meta-GGAs is also crucial for SAPT(DFT). Our results indicate that the older meta-GGAs, TPSS and revTPSS, benefit from the GRAC correction since we observe an improvement in total interaction energies as well as dramatic improvements in the energy components. In the newer meta-GGAs, MVS and SCAN, improvements in individual energy contributions are moderate but effectively add up to a notable reduction of errors in total interaction energies.

It is worth noting that the LC correction is fully compatible with meta-GGAs as our LC-meta-GGA is one of the best methods overall. The results could be further improved by tuning of the range-separation parameters specifically for LC-PBETPSS.

Electrostatic energy can serve as the most sensitive diagnostic tool in assessing electron densities of monomers. The quality of DFT densities has recently become a topic of intense literature debate concerning the DFT’s ability of accurately describing the energy and density simultaneously [48]. For both SCAN- and TPSS-based functionals, electrostatic energies agree with reference to within 5% or better which is an excellent result. The only meta-GGA which does not yield accurate densities is MVS. Also, in all the cases examined here, the DFT densities are superior to HF ones.

The first-order SAPT(DFT) exchange energies, which depend on the quality of the density matrix, appear to be slightly less accurate than electrostatics even upon the AC correction. However, SCAN0 yields some of the best exchange energies in both the first and second orders even without the AC correction.

Which method gives the best values of dispersion energy is also of prime importance to selecting the density functional approximation for SAPT(DFT). TPSS and revTPSS give excellent dispersion energies, provided that the AC correction is applied. The SCAN-based functionals moderately underestimate the dispersion energies leading to the relative errors below 10% regardless of AC. This is partially compensated by similar underestimation of the exchange-dispersion contribution. MVS is again an outlier with errors much larger in magnitude than the rest of meta-GGAs.

Our results show that meta-GGAs (with the exception of MVS) represent a definite progress in SAPT(DFT) compared to pure GGA, such as PBEAC, with their more consistent predictions of energy components. However, none of the meta-GGAs is better than the hybrid GGA approach PBE0AC. TPSS and revTPSS with AC come close to PBE0AC in predicting SAPT components, but the errors build up and the total energies are less accurate than those of SAPT(PBE0AC). On the positive note, we find that the only DFT functional which can be used in SAPT without an AC correction is SCAN0 thus providing the evidence of a good quality of both the orbitals and orbital energies. This is a clear improvement with respect to all GGAs studied in the literature. The long-range corrected meta-GGA, LC-PBETPSS, is a very reliable performer both in terms of total interaction energies and all the energy components, thus demonstrating that range-separation is a robust scheme of enforcing the correct asymptote of the exchange-correlation potential.

## Electronic supplementary material

ESM 1 (PDF 432 kb)
