# Supplementary material for: Assessment of SAPT(DFT) with meta-GGA functionals
Source: J Mol Model. 2020 Apr 15;26(5):102. doi: 10.1007/s00894-020-4340-9 (PMC7256086; doi:10.1007/s00894-020-4340-9)
Supplement: Supplementary file 1 — (PDF 432 kb) [file 894_2020_4340_MOESM1_ESM.pdf]

Supporting Information: Assesment of SAPT(DFT) with meta-GGA functionals

Michał Hapka,[*,†,‡] Marcin Modrzejewski,[‡] Grzegorz Chałasiński,[‡] and Małgorzata M. Szcześniak[*,¶]

†*Department of Chemistry, University of Michigan, Ann Arbor, Michigan 48109, USA*

‡*Faculty of Chemistry, University of Warsaw, ul. L. Pasteura 1, 02-093 Warsaw, Poland*

¶*Department of Chemistry, Oakland University, Rochester, Michigan 48309, USA*

E-mail: hapka@tiger.chem.uw.edu.pl; bryant@oakland.edu

List of Figures

S1 Relative percent errors of $E^{(1)}_{\rm elst}$ at SAPT2 and SAPT2+(3) levels of theory. . . S-5

S2 Relative percent errors of $E^{(1)}_{\rm elst}$ and $E^{(1)}_{\rm exch}$ SAPT energies (TK21 dataset). . . S-6

S3 Relative percent errors of $E^{(2)}_{\rm ind}$ and $E^{(2)}_{\rm exch-ind}$ SAPT energies (TK21 dataset). S-7

S4 Relative percent errors of $E^{(2)}_{\rm disp}$ and $E^{(2)}_{\rm exch-disp}$ SAPT energies (TK21 dataset). S-8

S5 Relative percent errors of $E^{\rm SAPT}_{\rm int}$ energies (TK21 dataset). S-25

List of Tables

S1 SAPT(CCSD) interaction energies and SAPT(DFT) relative percent errors (TK21 dataset, aug-cc-pVTZ). S-3

S2 CCSD(T), SAPT(CCSD) interaction energies with SAPT(PBE0AC) relative percent errors. S-4
S3 $E^{(1)}_{\mathrm{elst}}$: SAPT(CCSD) and SAPT(DFT) results (TK21, aug-cc-pVDZ). S-9
S4 $E^{(1)}_{\mathrm{exch}}(S^2)$: SAPT(CCSD) and SAPT(DFT) results (TK21, aug-cc-pVDZ). . . S-10
S5 $E^{(2)}_{\mathrm{ind}}$: SAPT(CCSD) and SAPT(DFT) results (TK21, aug-cc-pVDZ). S-11
S6 $E^{(2)}_{\mathrm{exch-ind}}(S^2)$: SAPT(CCSD) and SAPT(DFT) results (TK21, aug-cc-pVDZ). S-12
S7 $E^{(2)}_{\mathrm{disp}}$: SAPT(CCSD) and SAPT(DFT) results (TK21, aug-cc-pVDZ). S-13
S8 $E^{(2)}_{\mathrm{exch-disp}}(S^2)$: SAPT(CCSD) and SAPT(DFT) results (TK21, aug-cc-pVDZ). S-14
S9 $E^{\mathrm{SAPT}}_{\mathrm{int}}$: SAPT(CCSD) and SAPT(DFT) results (TK21, aug-cc-pVDZ). . . . S-15
S10 SAPT(PBE0AC) results (TK21, aug-cc-pVDZ). S-16
S11 $E^{(1)}_{\mathrm{elst}}$: SAPT(CCSD) and SAPT(DFT) results (TK21, aug-cc-pVTZ). S-17
S12 $E^{(1)}_{\mathrm{exch}}(S^2)$: SAPT(CCSD) and SAPT(DFT) results (TK21, aug-cc-pVTZ). . . S-18
S13 $E^{(2)}_{\mathrm{ind}}$: SAPT(CCSD) and SAPT(DFT) results (TK21, aug-cc-pVTZ). S-19
S14 $E^{(2)}_{\mathrm{exch-ind}}(S^2)$: SAPT(CCSD) and SAPT(DFT) results (TK21, aug-cc-pVTZ). S-20
S15 $E^{(2)}_{\mathrm{disp}}$: SAPT(CCSD) and SAPT(DFT) results (TK21, aug-cc-pVTZ). S-21
S16 $E^{(2)}_{\mathrm{exch-disp}}(S^2)$: SAPT(CCSD) and SAPT(DFT) results (TK21, aug-cc-pVTZ). S-22
S17 $E^{\mathrm{SAPT}}_{\mathrm{int}}$: SAPT(CCSD) and SAPT(DFT) results (TK21, aug-cc-pVTZ). . . . S-23
S18 SAPT(PBE0AC) results (TK21, aug-cc-pVTZ). S-24

Table S1: Reference SAPT(CCSD) interaction energies (in milliHartree) for the TK21/S_2 dataset and relative percent errors of SAPT(DFT). The basis set is aug-cc-pVTZ.

	CCSD	TPSS	TPSS AC	revTPSS	revTPSS AC	MVS	MVS AC	SCAN	SCAN AC	SCAN0	SCAN0 AC	LC-PBETPSS	SAPT2 +(CCD)
F^--HF	−66.94	11.83	12.47	14.95	15.58	2.60	2.58	9.96	9.87	−3.80	−3.62	−3.28	−10.52
F^--H_2O	−29.39	16.59	16.88	19.03	19.26	4.59	4.53	12.90	12.90	0.19	0.03	7.22	−9.58
Na^+-H_2O	−37.34	3.56	1.92	4.35	2.70	0.12	−0.36	1.66	0.76	−1.56	−2.01	−5.14	−1.96
HF-HF	−5.499	22.80	10.25	24.51	12.08	2.50	0.15	11.58	5.33	-2.37	−3.22	-10.98	-7.69
H_2O-H_2O	−6.223	23.88	14.83	25.64	16.79	1.50	1.62	13.81	10.87	0.36	0.31	-2.19	-6.66
NH_3-CH_4	−1.166	25.72	24.56	27.30	26.85	10.37	20.93	17.77	24.50	10.00	21.45	15.59	-2.25
NH_3-H_2O	−8.247	19.82	17.07	21.19	18.57	4.78	5.37	15.25	16.07	4.01	6.39	2.37	-3.35
CH_4-CH_4	−0.858	20.97	23.20	20.37	23.32	17.41	28.44	17.79	25.96	12.19	24.82	14.51	0.79
N_2-N_2	−0.464	23.54	4.82	22.79	4.65	15.60	10.26	19.77	10.59	16.13	14.97	15.47	−4.29
P_2-P_2	−1.599	21.62	16.01	21.48	16.25	22.05	26.63	21.36	22.16	18.94	20.19	26.23	32.34
N_2O-He(GM)	−0.247	38.61	12.52	37.35	13.37	33.62	20.38	33.51	15.15	20.79	10.86	2.73	−3.36
N_2O-He(LM)	−0.135	44.29	14.86	41.64	14.32	37.49	23.51	35.92	16.20	21.00	10.94	10.96	−3.19
CO_2-He(GM)	−0.186	54.60	14.98	53.05	15.47	43.00	24.47	45.06	18.27	25.87	13.71	2.34	−4.66
CO_2-He(LM)	−0.110	42.48	10.98	40.38	10.82	35.09	20.85	34.17	13.56	19.64	10.62	10.08	−4.65
Ar-Ar	−0.336	16.75	2.41	16.73	2.55	17.17	13.69	15.71	7.96	11.41	7.59	9.24	0.74
Mean % error		25.80	13.19	26.05	14.17	16.53	13.53	20.41	14.01	10.19	8.87	6.34	−1.89
MAE		25.80	13.19	26.05	14.17	16.53	13.58	20.41	14.01	11.22	10.05	9.22	6.40

Table S2: Reference CCSD(T) and SAPT(CCSD) interaction energies (in milliHartree) for the TK21 dataset and relative percent errors of SAPT(PBE0AC) [with respect to SAPT(CCSD)] in the aug-cc-pVXZ (X=D,T) basis sets.

	$E_{\text{int}}^{\text{CCSD(T)}}$		$E_{\text{int}}^{\text{SAPT(CCSD)}}$		SAPT(PBE0AC)	
	AVDZ(S_1)	AVTZ(S_2)	AVDZ(S_1)	AVTZ(S_2)	AVDZ(S_1)	AVTZ(S_2)
F^--HF	−95.37	−100.9	−58.98	−66.94	−6.52	−0.29
F^--H_2O	−36.15	−38.51	−25.59	−29.39	−0.51	4.86
Na^+-H_2O	−35.01	−35.90	−36.19	−37.34	−0.84	−0.41
HF-HF	−6.198	−6.670	−4.783	−5.500	−4.37	0.95
H_2O-H_2O	−6.854	−7.499	−5.256	−6.220	−1.85	4.60
NH_3-CH_4	−0.898	−1.107	−0.907	−1.166	16.43	19.3
NH_3-H_2O	−8.921	−9.869	−6.919	−8.247	3.43	9.09
C_2H_6-HCN	−1.355		−1.220		2.95	
CH_4-CH_4	−0.674	−0.787	−0.717	−0.858	24.27	22.4
C_2H_2-C_2H_2(PD)	−1.731		−1.715		−5.25	
C_2H_2-C_2H_2(S)	−0.128		−0.208		26.44	
C_2H_2-C_2H_2(T)	−1.902		−1.741		−9.82	
NCCN-NCCN	−2.737		−2.728		13.78	
PCCP-PCCP	−2.576		−3.177		16.93	
N_2-N_2	−0.350	−0.427	−0.381	−0.464	11.29	10.8
P_2-P_2	−0.653	−1.154	−1.010	−1.599	11.88	15.4
N_2O-He(GM)	−0.151	−0.235	−0.155	−0.247	1.29	4.86
N_2O-He(LM)	−0.089	−0.131	−0.089	−0.135	6.74	5.19
CO_2-He(GM)	−0.103	−0.179	−0.102	−0.186	2.94	9.68
CO_2-He(LM)	−0.071	−0.107	−0.070	−0.110	4.29	6.36
Ar-Ar	−0.196	−0.317	−0.203	−0.336	−2.46	3.27
Mean % error					5.29	7.73
MAE					8.30	7.82

CCSD(T) results taken from T. Korona, Mol. Phys. 111:3705-3715 (2013)

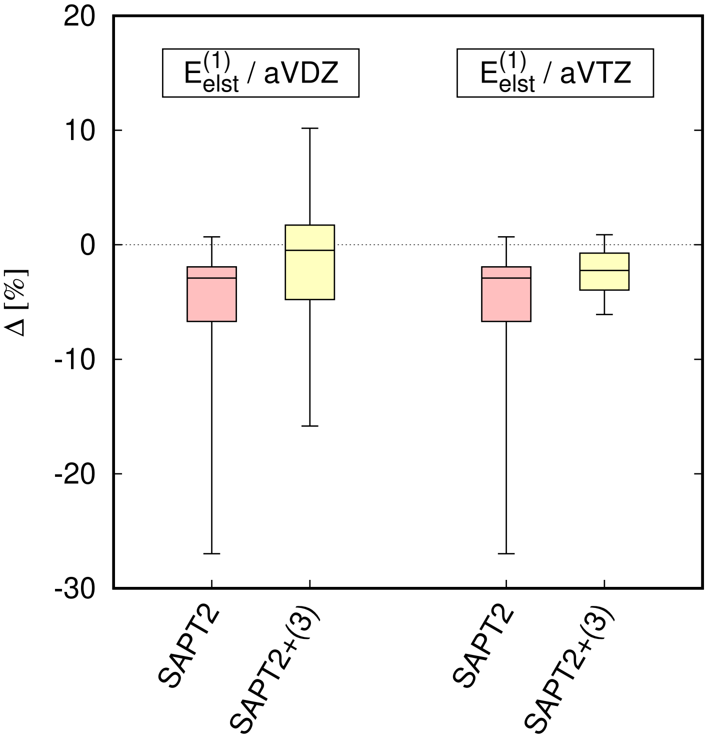

Figure S1: Box plot of relative percent errors of electrostatic energy ($E^{(1)}_{\mathrm{elst}}$) for dimers of the TK21 data set calculated at the SAPT2 ($E^{(1)}_{\mathrm{elst}} = E^{(10)}_{\mathrm{elst}} + E^{(12)}_{\mathrm{elst}}$) and SAPT2+(3) ($E^{(1)}_{\mathrm{elst}} = E^{(10)}_{\mathrm{elst}} + E^{(12)}_{\mathrm{elst}} + E^{(13)}_{\mathrm{elst}}$) levels of theory. Errors with respect to SAPT(CCSD) results. The box and outer fences encompass 50% and 100% of the distribution, respectively.

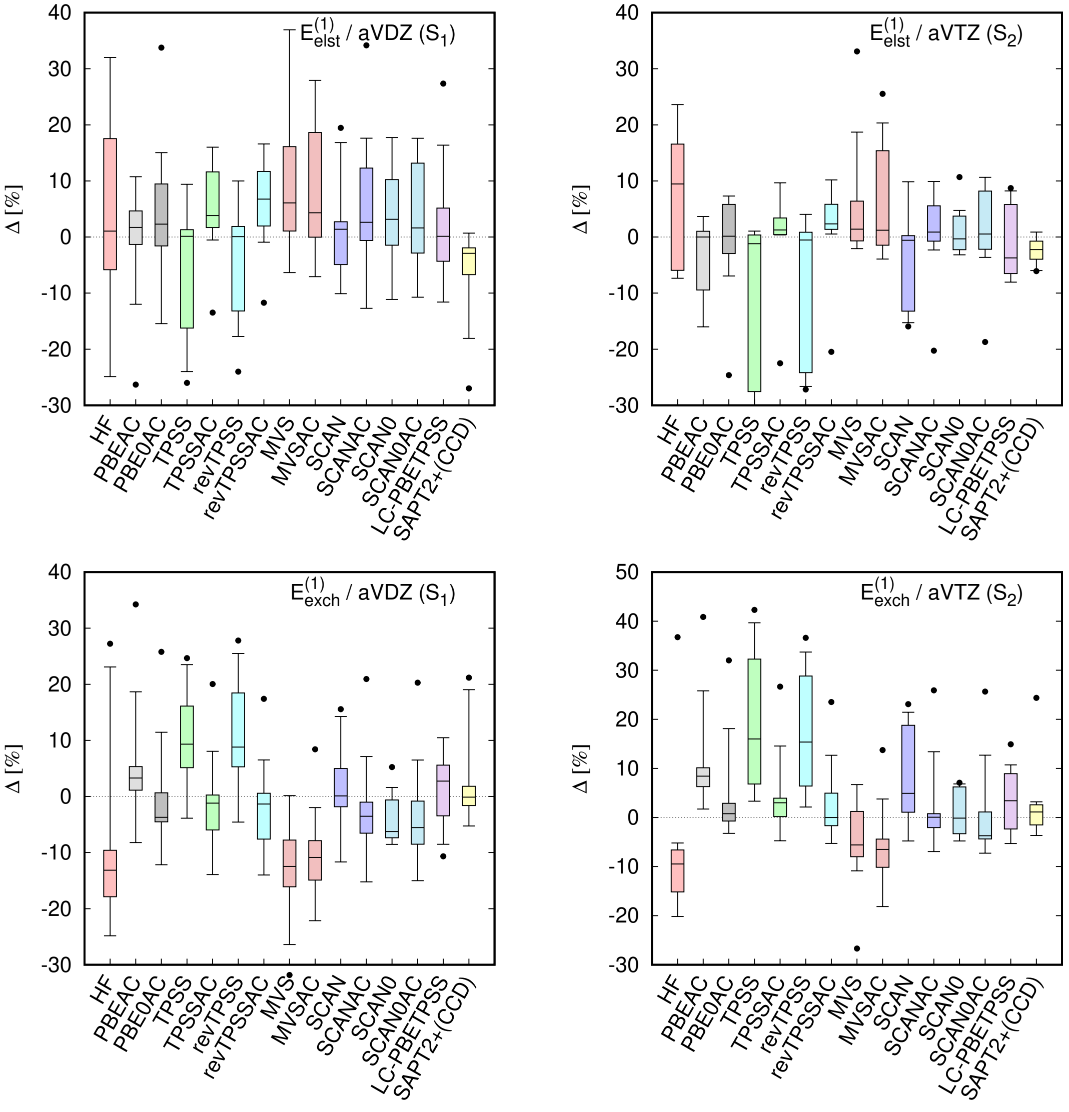

Figure S2: Box plot of relative percent errors of electrostatic ($E^{(1)}_{\mathrm{elst}}$) and first-order exchange ($E^{(1)}_{\mathrm{exch}}$) SAPT energies for dimers of the TK21 data set calculated in aug-cc-pVDZ (left) and aug-cc-pVTZ (right) basis sets. Errors with respect to SAPT(CCSD) results. Single outliers are represented by dots.

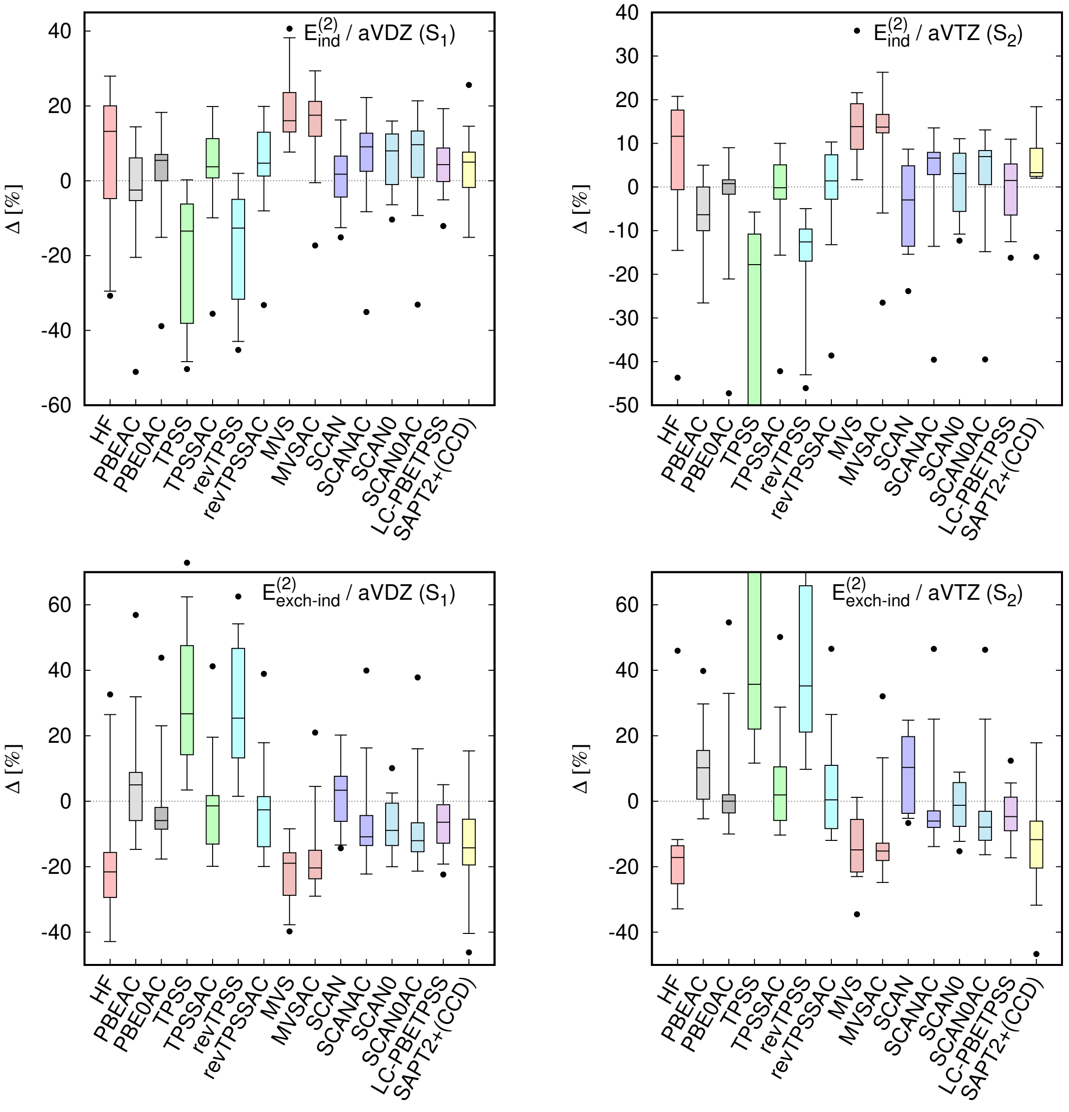

Figure S3: Box plot of relative percent errors of second-order induction and exchange-inductions energies for dimers of the TK21 data set calculated in aug-cc-pVDZ (left) and aug-cc-pVTZ (right) basis sets. The δ_{HF} term is not included. Errors with respect to SAPT(CCSD) results.

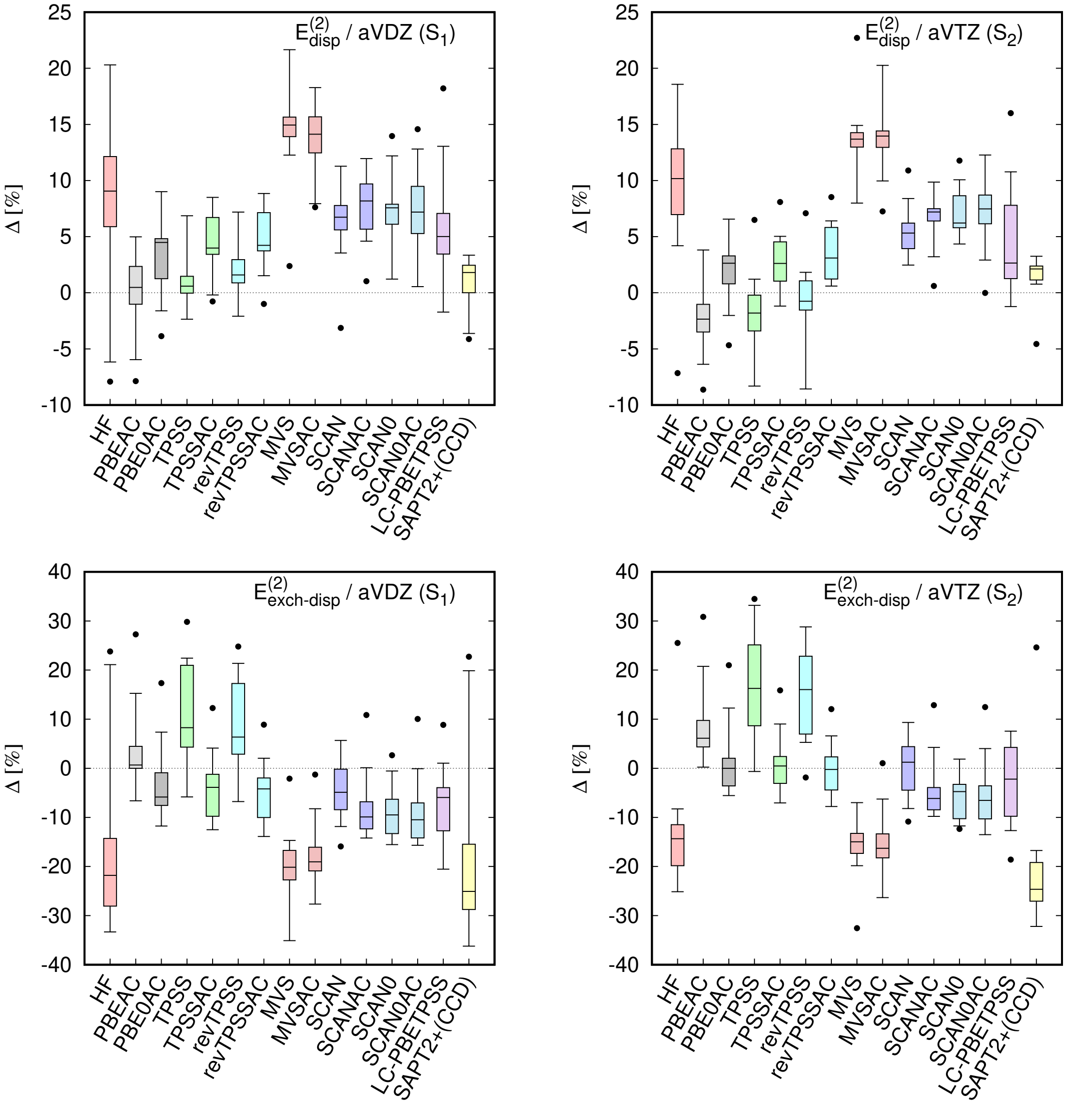

Figure S4: Box plot of relative percent errors of second-order dispersion and exchange-dispersion energies for dimers of the TK21 data set calculated in aug-cc-pVDZ (left) and aug-cc-pVTZ (right) basis sets. Errors with respect to SAPT(CCSD) results.

Table S3: First order electrostatic energy (in milliHartree) on the TK21/S1 dataset from SAPT(CCSD) and SAPT(DFT) calculations. The basis set is aug-cc-pVDZ.

$E^{(1)}_{\mathrm{elst}}$	CCSD	TPSS	TPSS AC	revTPSS	revTPSS AC	MVS	MVS AC	SCAN	SCAN AC	SCAN0	SCAN0 AC	LC-PBETPSS	SAPT2
F^--HF	-114.4	-114.3	-113.6	-114.3	-113.7	-116.9	-116.7	-115.5	-115.1	-117.1	-116.9	-115.7	-114.9
F^--H_2O	-61.87	-61.10	-60.45	-61.11	-60.45	-60.49	-60.40	-61.02	-60.69	-60.95	-60.87	-61.80	-62.11
Na^+-H_2O	-39.34	-38.95	-38.74	-38.78	-38.56	-40.25	-40.33	-39.72	-39.69	-40.54	-40.61	-42.49	-40.11
HF-HF	-9.569	-9.534	-9.407	-9.506	-9.380	-9.47	-9.474	-9.593	-9.563	-9.709	-9.731	-10.13	-9.782
H_2O-H_2O	-13.31	-13.08	-12.80	-13.06	-12.79	-13.04	-13.02	-13.11	-13.00	-13.19	-13.19	-13.78	-0.296
NH_3-CH_4	-0.937	-0.925	-0.918	-0.891	-0.889	-0.880	-0.937	-0.921	-0.959	-0.911	-0.971	-0.970	-13.60
NH_3-H_2O	-18.10	-17.78	-17.41	-17.84	-17.48	-17.87	-17.79	-17.81	-17.63	-17.86	-17.81	-18.60	-0.971
C_2H_6-HCN	-1.026	-1.023	-0.999	-0.978	-0.957	-1.015	-1.038	-1.018	-1.027	-1.043	-1.055	-1.088	-18.48
CH_4-CH_4	-0.285	-0.310	-0.323	-0.302	-0.318	-0.216	-0.273	-0.278	-0.321	-0.256	-0.315	-0.270	-0.271
C_2H_2-C_2H_2(PD)	-2.334	-2.429	-2.322	-2.425	-2.321	-2.476	-2.499	-2.476	-2.445	-2.571	-2.560	-2.524	-2.402
C_2H_2-C_2H_2(S)	0.154	0.114	0.178	0.117	0.180	0.292	0.282	0.184	0.207	0.220	0.229	0.196	0.126
C_2H_2-C_2H_2(T)	-2.600	-2.676	-2.614	-2.684	-2.624	-2.765	-2.766	-2.773	-2.751	-2.889	-2.879	-2.902	-2.650
NCCN-NCCN	-3.524	-3.382	-3.301	-3.347	-3.274	-3.059	-3.198	-3.319	-3.373	-3.262	-3.333	-3.676	-1.095
PCCP-PCCP	-3.144	-2.848	-2.778	-2.830	-2.776	-2.144	-2.457	-2.667	-2.828	-2.779	-2.907	-3.022	-3.795
N_2-N_2	-0.267	-0.281	-0.245	-0.278	-0.244	-0.224	-0.219	-0.246	-0.232	-0.220	-0.221	-0.239	-1.136
P_2-P_2	-1.068	-0.982	-0.897	-0.974	-0.899	-0.673	-0.770	-0.888	-0.915	-0.938	-0.971	-1.050	-0.095
N_2O-He(GM)	-0.095	-0.110	-0.084	-0.108	-0.083	-0.087	-0.077	-0.100	-0.083	-0.092	-0.082	-0.084	-0.041
N_2O-He(LM)	-0.036	-0.044	-0.033	-0.042	-0.032	-0.034	-0.031	-0.039	-0.033	-0.034	-0.031	-0.034	-0.086
CO_2-He(GM)	-0.087	-0.102	-0.077	-0.100	-0.076	-0.078	-0.068	-0.091	-0.076	-0.082	-0.074	-0.075	-0.032
CO_2-He(LM)	-0.025	-0.031	-0.023	-0.029	-0.022	-0.023	-0.022	-0.028	-0.023	-0.024	-0.022	-0.024	-0.097
Ar-Ar	-0.093	-0.109	-0.078	-0.108	-0.079	-0.074	-0.070	-0.090	-0.077	-0.082	-0.077	-0.078	-3.359

Table S4: First order exchange energy in the S^2 approximation (in milliHartree) on the TK21/S1 dataset from SAPT(CCSD) and SAPT(DFT) calculations. The basis set is aug-cc-pVDZ.

$E^{(1)}_{\mathrm{exch}}(S^2)$	CCSD	TPSS	TPSS AC	revTPSS	revTPSS AC	MVS	MVS AC	SCAN	SCAN AC	SCAN0	SCAN0 AC	LC-PBETPSS	SAPT2
F^--HF	127.6	128.1	127.9	129.8	129.6	117.7	117.5	124.1	124.0	117.4	117.2	116.7	129.4
F^--H_2O	63.84	64.58	64.01	65.10	64.49	58.20	58.13	62.31	62.05	58.74	58.69	61.64	64.99
Na^+-H_2O	14.57	15.38	14.26	15.34	14.22	13.59	13.37	14.47	13.95	13.93	13.78	15.42	14.51
HF-HF	9.181	10.15	9.219	10.15	9.233	8.358	8.201	9.207	8.796	8.487	8.400	8.569	9.169
H_2O-H_2O	13.73	14.48	13.57	14.49	13.61	12.29	12.25	13.48	13.16	12.66	12.66	13.27	13.81
NH_3-CH_4	1.774	1.951	1.917	1.906	1.890	1.519	1.739	1.763	1.900	1.659	1.889	1.833	1.766
NH_3-H_2O	19.05	19.59	18.86	19.70	19.01	17.38	17.56	18.70	18.56	17.83	17.99	18.81	19.36
C_2H_6-HCN	1.990	2.195	2.091	2.154	2.067	1.742	1.917	1.992	2.064	1.878	2.002	1.995	1.958
CH_4-CH_4	0.981	1.134	1.178	1.095	1.152	0.858	1.064	1.030	1.186	0.961	1.180	1.017	0.976
C_2H_2-C_2H_2(PD)	2.712	3.055	2.706	3.009	2.676	2.267	2.405	2.772	2.711	2.695	2.695	2.872	2.921
C_2H_2-C_2H_2(S)	0.628	0.786	0.602	0.783	0.603	0.516	0.529	0.680	0.606	0.661	0.625	0.694	0.748
C_2H_2-C_2H_2(T)	3.077	3.368	3.041	3.348	3.038	2.610	2.742	3.105	3.046	3.052	3.052	3.321	3.289
NCCN-NCCN	3.988	4.277	4.039	4.230	4.011	3.346	3.706	3.896	4.028	3.695	3.880	4.194	3.947
PCCP-PCCP	7.095	6.953	6.811	6.902	6.801	5.223	6.037	6.512	6.936	6.651	6.981	7.491	8.098
N_2-N_2	0.544	0.646	0.491	0.636	0.490	0.468	0.438	0.553	0.488	0.508	0.507	0.539	0.515
P_2-P_2	2.722	2.620	2.418	2.598	2.419	1.856	2.126	2.404	2.488	2.530	2.623	2.897	3.299
N_2O-He(GM)	0.405	0.505	0.379	0.490	0.374	0.389	0.347	0.455	0.378	0.411	0.370	0.376	0.393
N_2O-He(LM)	0.193	0.252	0.185	0.242	0.180	0.193	0.172	0.221	0.181	0.194	0.174	0.198	0.188
CO_2-He(GM)	0.404	0.514	0.380	0.498	0.373	0.389	0.341	0.457	0.376	0.406	0.364	0.371	0.393
CO_2-He(LM)	0.149	0.198	0.140	0.190	0.137	0.149	0.131	0.172	0.139	0.149	0.134	0.153	0.141
Ar-Ar	0.304	0.362	0.262	0.360	0.261	0.248	0.237	0.304	0.258	0.278	0.258	0.272	0.305

Table S5: Second order induction energy (in milliHartree) on the TK21/S1 dataset from SAPT(CCSD) and SAPT(DFT) calculations. The basis set is aug-cc-pVDZ.

$E_{\text{ind}}^{(2)}$	CCSD	TPSS	TPSS AC	revTPSS	revTPSS AC	MVS	MVS AC	SCAN	SCAN AC	SCAN0	SCAN0 AC	LC-PBETPSS	SAPT2
F^--HF	-109.9	-111.9	-109.0	-112.1	-109.2	-90.52	-89.43	-99.74	-98.17	-95.40	-94.71	-95.25	-106.0
F^--H_2O	-46.91	-49.83	-47.45	-49.62	-47.23	-39.43	-38.70	-44.21	-42.97	-40.87	-40.54	-41.38	-44.05
Na^+-H_2O	-25.48	-26.40	-24.42	-26.06	-24.16	-21.84	-21.65	-23.81	-23.07	-23.12	-23.02	-24.38	-24.89
HF-HF	-4.627	-5.249	-4.518	-5.213	-4.493	-4.042	-3.892	-4.531	-4.201	-4.211	-4.132	-4.221	-4.430
H_2O-H_2O	-5.940	-6.533	-5.717	-6.477	-5.682	-4.878	-4.770	-5.549	-5.236	-5.197	-5.149	-5.397	-5.620
NH_3-CH_4	-0.430	-0.482	-0.473	-0.468	-0.465	-0.366	-0.432	-0.422	-0.466	-0.396	-0.470	-0.419	-0.409
NH_3-H_2O	-9.366	-9.889	-9.120	-9.830	-9.099	-7.343	-7.464	-8.281	-8.174	-8.039	-8.209	-8.818	-8.927
C_2H_6-HCN	-0.841	-0.901	-0.876	-0.883	-0.864	-0.777	-0.830	-0.848	-0.877	-0.851	-0.898	-0.873	-0.856
CH_4-CH_4	-0.139	-0.179	-0.188	-0.172	-0.185	-0.121	-0.163	-0.154	-0.188	-0.136	-0.185	-0.132	-0.128
C_2H_2-C_2H_2(PD)	-0.763	-0.936	-0.751	-0.923	-0.745	-0.641	-0.672	-0.797	-0.744	-0.780	-0.761	-0.765	-0.834
C_2H_2-C_2H_2(S)	-0.183	-0.254	-0.167	-0.254	-0.168	-0.161	-0.152	-0.211	-0.168	-0.202	-0.179	-0.192	-0.211
C_2H_2-C_2H_2(T)	-0.885	-1.006	-0.878	-0.997	-0.874	-0.762	-0.785	-0.902	-0.865	-0.912	-0.898	-0.930	-0.977
NCCN-NCCN	-1.425	-1.520	-1.360	-1.492	-1.343	-1.089	-1.238	-1.288	-1.329	-1.197	-1.273	-1.415	-1.274
PCCP-PCCP	-3.720	-3.711	-3.450	-3.646	-3.429	-2.298	-2.931	-3.116	-3.413	-3.180	-3.417	-3.536	-4.020
N_2-N_2	-0.093	-0.123	-0.079	-0.121	-0.079	-0.082	-0.069	-0.097	-0.077	-0.082	-0.079	-0.082	-0.079
P_2-P_2	-1.428	-1.488	-1.211	-1.463	-1.209	-0.847	-1.008	-1.213	-1.214	-1.261	-1.294	-1.419	-1.624
N_2O-He(GM)	-0.050	-0.070	-0.043	-0.067	-0.042	-0.035	-0.039	-0.050	-0.043	-0.048	-0.043	-0.047	-0.044
N_2O-He(LM)	-0.013	-0.019	-0.011	-0.018	-0.011	-0.008	-0.010	-0.013	-0.011	-0.013	-0.011	-0.014	-0.010
CO_2-He(GM)	-0.049	-0.068	-0.043	-0.065	-0.043	-0.036	-0.040	-0.051	-0.045	-0.049	-0.046	-0.048	-0.046
CO_2-He(LM)	-0.010	-0.015	-0.010	-0.014	-0.010	-0.008	-0.009	-0.011	-0.010	-0.011	-0.010	-0.011	-0.009
Ar-Ar	-0.104	-0.152	-0.083	-0.151	-0.083	-0.091	-0.074	-0.117	-0.081	-0.099	-0.082	-0.084	-0.097

Table S6: Second order exchange-induction energy (in milliHartree) on the TK21/S1 dataset from SAPT(CCSD) and SAPT(DFT) calculations. The basis set is aug-cc-pVDZ.

$E^{(2)}_{\mathrm{exch-ind}}(S^2)$	CCSD	TPSS	TPSS AC	revTPSS	revTPSS AC	MVS	MVS AC	SCAN	SCAN AC	SCAN0	SCAN0 AC	LC-PBETPSS	SAPT2
F^--HF	51.69	56.06	53.80	56.39	54.08	39.41	38.60	46.58	45.40	41.36	40.86	40.12	38.85
F^--H_2O	27.77	32.08	29.87	31.95	29.71	22.83	22.11	26.95	25.78	23.18	22.87	23.64	21.30
Na^+-H_2O	14.39	15.50	13.82	15.24	13.63	12.13	12.01	13.51	12.92	12.91	12.86	14.29	13.60
HF-HF	2.493	3.204	2.490	3.184	2.482	2.190	2.042	2.578	2.258	2.235	2.156	2.116	2.023
H_2O-H_2O	3.672	4.460	3.627	4.420	3.609	2.977	2.869	3.551	3.238	3.154	3.105	3.194	3.076
NH_3-CH_4	0.282	0.349	0.337	0.338	0.332	0.234	0.295	0.289	0.328	0.257	0.327	0.267	0.242
NH_3-H_2O	5.852	6.684	5.953	6.627	5.935	4.311	4.466	5.196	5.131	4.864	5.064	5.372	5.110
C_2H_6-HCN	0.313	0.390	0.365	0.381	0.361	0.270	0.317	0.324	0.348	0.295	0.339	0.294	0.256
CH_4-CH_4	0.130	0.175	0.184	0.168	0.181	0.115	0.157	0.149	0.182	0.130	0.179	0.125	0.120
C_2H_2-C_2H_2(PD)	0.590	0.787	0.590	0.774	0.585	0.477	0.502	0.635	0.574	0.603	0.579	0.587	0.634
C_2H_2-C_2H_2(S)	0.136	0.210	0.122	0.210	0.123	0.112	0.104	0.163	0.121	0.150	0.127	0.142	0.153
C_2H_2-C_2H_2(T)	0.553	0.701	0.557	0.693	0.555	0.449	0.468	0.582	0.537	0.567	0.549	0.581	0.607
NCCN-NCCN	1.030	1.177	1.015	1.154	1.003	0.796	0.931	0.955	0.985	0.856	0.922	1.019	0.835
PCCP-PCCP	3.409	3.526	3.260	3.461	3.237	2.123	2.743	2.920	3.209	2.948	3.178	3.298	3.712
N_2-N_2	0.082	0.115	0.070	0.113	0.070	0.075	0.062	0.090	0.069	0.075	0.072	0.074	0.072
P_2-P_2	1.342	1.432	1.158	1.407	1.156	0.809	0.962	1.163	1.161	1.205	1.235	1.359	1.549
N_2O-He(GM)	0.033	0.054	0.028	0.051	0.028	0.024	0.026	0.035	0.028	0.033	0.028	0.029	0.027
N_2O-He(LM)	0.010	0.017	0.009	0.016	0.009	0.007	0.009	0.011	0.009	0.010	0.009	0.009	0.005
CO_2-He(GM)	0.030	0.048	0.026	0.045	0.026	0.021	0.024	0.032	0.026	0.029	0.026	0.026	0.022
CO_2-He(LM)	0.007	0.011	0.006	0.010	0.006	0.005	0.006	0.007	0.006	0.006	0.006	0.006	0.004
Ar-Ar	0.102	0.151	0.082	0.150	0.082	0.090	0.072	0.116	0.079	0.098	0.080	0.082	0.095

Table S7: Second order dispersion energy (in milliHartree) on the TK21/S1 dataset from SAPT(CCSD) and SAPT(DFT) calculations. The basis set is aug-cc-pVDZ.

$E^{(2)}_{\mathrm{disp}}$	CCSD	TPSS	TPSS AC	revTPSS	revTPSS AC	MVS	MVS AC	SCAN	SCAN AC	SCAN0	SCAN0 AC	LC -PBETPSS	SAPT2 +(CCD)
F^--HF	-21.53	-20.73	-20.29	-20.75	-20.31	-17.86	-17.67	-19.23	-18.96	-18.53	-18.39	-17.61	-21.35
F^--H_2O	-13.30	-13.32	-12.83	-13.28	-12.80	-11.27	-11.10	-12.27	-12.00	-11.68	-11.60	-11.56	-12.97
Na^+-H_2O	-0.436	-0.502	-0.439	-0.504	-0.440	-0.426	-0.401	-0.450	-0.415	-0.431	-0.413	-0.444	-0.438
HF-HF	-2.713	-2.751	-2.617	-2.745	-2.613	-2.321	-2.294	-2.514	-2.452	-2.422	-2.405	-2.397	-2.664
H_2O-H_2O	-4.307	-4.282	-4.113	-4.264	-4.101	-3.632	-3.609	-3.939	-3.872	-3.838	-3.827	-3.873	-4.217
NH_3-CH_4	-1.786	-1.761	-1.752	-1.733	-1.729	-1.534	-1.593	-1.667	-1.704	-1.651	-1.709	-1.689	-1.737
NH_3-H_2O	-5.708	-5.635	-5.480	-5.617	-5.470	-4.794	-4.813	-5.187	-5.155	-5.105	-5.132	-5.258	-5.599
C_2H_6-HCN	-1.807	-1.778	-1.755	-1.753	-1.733	-1.572	-1.614	-1.685	-1.705	-1.670	-1.697	-1.696	-1.746
CH_4-CH_4	-1.525	-1.510	-1.528	-1.480	-1.502	-1.338	-1.409	-1.455	-1.509	-1.448	-1.517	-1.470	-1.485
C_2H_2-C_2H_2(PD)	-2.190	-2.191	-2.117	-2.171	-2.101	-1.861	-1.904	-2.077	-2.073	-2.093	-2.101	-2.114	-2.192
C_2H_2-C_2H_2(S)	-1.046	-1.071	-1.008	-1.068	-1.007	-0.889	-0.905	-1.009	-0.991	-1.027	-1.021	-1.017	-1.066
C_2H_2-C_2H_2(T)	-2.154	-2.143	-2.080	-2.129	-2.070	-1.817	-1.851	-2.029	-2.025	-2.049	-2.055	-2.098	-2.154
NCCN-NCCN	-3.178	-3.094	-3.052	-3.082	-3.044	-2.708	-2.786	-2.940	-2.975	-2.927	-2.971	-3.019	-3.141
PCCP-PCCP	-7.927	-7.396	-7.367	-7.377	-7.361	-6.413	-6.673	-7.137	-7.279	-7.329	-7.440	-7.471	-8.254
N_2-N_2	-0.693	-0.683	-0.649	-0.680	-0.649	-0.598	-0.598	-0.641	-0.632	-0.639	-0.643	-0.653	-0.675
P_2-P_2	-2.971	-2.767	-2.719	-2.758	-2.716	-2.328	-2.428	-2.636	-2.677	-2.742	-2.781	-2.849	-3.079
N_2O-He(GM)	-0.468	-0.468	-0.436	-0.461	-0.432	-0.401	-0.402	-0.443	-0.430	-0.439	-0.432	-0.450	-0.458
N_2O-He(LM)	-0.252	-0.249	-0.232	-0.245	-0.230	-0.214	-0.214	-0.234	-0.227	-0.232	-0.228	-0.245	-0.249
CO_2-He(GM)	-0.417	-0.419	-0.389	-0.413	-0.385	-0.352	-0.352	-0.394	-0.381	-0.386	-0.379	-0.398	-0.407
CO_2-He(LM)	-0.197	-0.197	-0.184	-0.194	-0.182	-0.170	-0.169	-0.185	-0.179	-0.182	-0.179	-0.193	-0.193
Ar-Ar	-0.433	-0.432	-0.411	-0.431	-0.410	-0.378	-0.379	-0.405	-0.398	-0.402	-0.400	-0.402	-0.431

Table S8: Second order exchange-dispersion energy in the S^2 approximation (in milliHartree) on the TK21/S1 dataset from SAPT(CCSD) and SAPT(DFT) calculations. The basis set is aug-cc-pVDZ.

$E^{(2)}_{\mathrm{exch-disp}}(S^2)$	CCSD	TPSS	TPSS AC	revTPSS	revTPSS AC	MVS	MVS AC	SCAN	SCAN AC	SCAN0	SCAN0 AC	LC-PBETPSS	SAPT2
F^--HF	7.543	7.828	7.451	7.822	7.443	6.292	6.141	6.996	6.777	6.460	6.361	5.994	5.574
F^--H_2O	4.887	5.238	4.914	5.208	4.887	4.130	4.023	4.642	4.469	4.237	4.187	4.143	3.509
Na^+-H_2O	0.101	0.122	0.103	0.123	0.103	0.099	0.093	0.106	0.097	0.100	0.096	0.110	0.085
HF-HF	0.452	0.511	0.444	0.509	0.443	0.386	0.371	0.438	0.407	0.399	0.390	0.381	0.322
H_2O-H_2O	0.893	0.952	0.856	0.946	0.853	0.727	0.713	0.822	0.784	0.770	0.763	0.775	0.669
NH_3-CH_4	0.190	0.201	0.198	0.195	0.194	0.153	0.172	0.178	0.190	0.170	0.190	0.179	0.138
NH_3-H_2O	1.353	1.388	1.301	1.381	1.298	1.046	1.059	1.193	1.176	1.144	1.161	1.195	1.056
C_2H_6-HCN	0.151	0.158	0.150	0.154	0.147	0.119	0.130	0.136	0.141	0.128	0.135	0.129	0.105
CH_4-CH_4	0.121	0.131	0.136	0.126	0.132	0.101	0.119	0.119	0.134	0.113	0.133	0.115	0.085
C_2H_2-C_2H_2(PD)	0.270	0.292	0.260	0.287	0.257	0.208	0.218	0.257	0.250	0.252	0.251	0.254	0.255
C_2H_2-C_2H_2(S)	0.103	0.123	0.099	0.122	0.099	0.082	0.084	0.106	0.097	0.106	0.101	0.103	0.112
C_2H_2-C_2H_2(T)	0.268	0.280	0.252	0.277	0.251	0.200	0.209	0.245	0.240	0.243	0.242	0.255	0.247
NCCN-NCCN	0.381	0.390	0.369	0.386	0.366	0.294	0.321	0.345	0.355	0.329	0.343	0.359	0.317
PCCP-PCCP	1.110	1.096	1.070	1.086	1.066	0.798	0.899	0.999	1.051	1.034	1.075	1.094	1.362
N_2-N_2	0.046	0.052	0.041	0.051	0.041	0.038	0.036	0.044	0.040	0.041	0.041	0.043	0.037
P_2-P_2	0.393	0.370	0.344	0.366	0.343	0.255	0.284	0.330	0.339	0.351	0.361	0.381	0.471
N_2O-He(GM)	0.020	0.024	0.018	0.023	0.018	0.016	0.016	0.020	0.018	0.019	0.018	0.018	0.015
N_2O-He(LM)	0.009	0.011	0.008	0.010	0.008	0.007	0.007	0.009	0.008	0.008	0.008	0.009	0.006
CO_2-He(GM)	0.017	0.021	0.015	0.020	0.015	0.013	0.013	0.017	0.015	0.016	0.014	0.015	0.011
CO_2-He(LM)	0.006	0.008	0.006	0.007	0.005	0.005	0.005	0.006	0.005	0.006	0.005	0.006	0.004
Ar-Ar	0.021	0.025	0.019	0.025	0.019	0.017	0.017	0.021	0.018	0.020	0.018	0.018	0.018

Table S9: Interaction energies on the TK21/S1 dataset from SAPT(CCSD) and SAPT(DFT) calculations. The basis set is aug-cc-pVDZ.

$E_{\text{int}}^{\text{SAPT}}$	CCSD	TPSS	TPSS AC	revTPSS	revTPSS AC	MVS	MVS AC	SCAN	SCAN AC	SCAN0	SCAN0 AC	LC-PBETPSS	SAPT2
F^--HF	-58.98	-54.94	-53.79	-53.17	-52.06	-61.85	-61.54	-56.73	-56.05	-65.78	-65.59	-65.75	-68.37
F^--H_2O	-25.59	-22.34	-21.95	-21.75	-21.40	-26.04	-25.93	-23.59	-23.36	-27.33	-27.26	-25.32	-29.33
Na^+-H_2O	-36.19	-34.85	-35.42	-34.64	-35.22	-36.70	-36.91	-35.89	-36.21	-37.15	-37.31	-37.49	-37.24
HF-HF	-4.783	-3.665	-4.388	-3.618	-4.328	-4.904	-5.047	-4.414	-4.754	-5.221	-5.322	-5.683	-5.363
H_2O-H_2O	-5.256	-4.004	-4.578	-3.943	-4.499	-5.564	-5.567	-4.743	-4.930	-5.643	-5.642	-5.815	-5.878
NH_3-CH_4	-0.907	-0.666	-0.691	-0.652	-0.667	-0.874	-0.757	-0.780	-0.710	-0.871	-0.744	-0.799	-0.972
NH_3-H_2O	-6.919	-5.639	-5.892	-5.577	-5.809	-7.275	-6.987	-6.186	-6.085	-7.161	-6.929	-7.294	-7.482
C_2H_6-HCN	-1.220	-0.959	-1.024	-0.925	-0.978	-1.232	-1.119	-1.100	-1.056	-1.264	-1.175	-1.238	-1.378
CH_4-CH_4	-0.717	-0.559	-0.543	-0.564	-0.541	-0.600	-0.504	-0.589	-0.516	-0.635	-0.524	-0.616	-0.727
C_2H_2-C_2H_2(PD)	-1.715	-1.421	-1.634	-1.447	-1.650	-2.026	-1.950	-1.687	-1.726	-1.895	-1.897	-1.691	-1.619
C_2H_2-C_2H_2(S)	-0.208	-0.092	-0.174	-0.090	-0.171	-0.048	-0.059	-0.086	-0.129	-0.092	-0.117	-0.074	-0.138
C_2H_2-C_2H_2(T)	-1.741	-1.476	-1.722	-1.492	-1.724	-2.085	-1.982	-1.771	-1.818	-1.989	-1.990	-1.772	-1.638
NCCN-NCCN	-2.728	-2.153	-2.289	-2.152	-2.280	-2.419	-2.263	-2.351	-2.309	-2.506	-2.432	-2.538	-3.111
PCCP-PCCP	-3.177	-2.380	-2.454	-2.405	-2.461	-2.712	-2.382	-2.488	-2.325	-2.655	-2.531	-2.146	-2.460
N_2-N_2	-0.381	-0.274	-0.370	-0.279	-0.370	-0.323	-0.349	-0.298	-0.344	-0.316	-0.322	-0.319	-0.401
P_2-P_2	-1.010	-0.815	-0.907	-0.823	-0.906	-0.929	-0.833	-0.840	-0.819	-0.854	-0.827	-0.682	-0.520
N_2O-He(GM)	-0.155	-0.065	-0.137	-0.071	-0.138	-0.095	-0.129	-0.082	-0.132	-0.116	-0.142	-0.158	-0.163
N_2O-He(LM)	-0.089	-0.031	-0.074	-0.036	-0.076	-0.050	-0.068	-0.046	-0.073	-0.067	-0.081	-0.077	-0.100
CO_2-He(GM)	-0.102	-0.007	-0.088	-0.013	-0.089	-0.043	-0.083	-0.029	-0.085	-0.067	-0.094	-0.110	-0.113
CO_2-He(LM)	-0.070	-0.026	-0.065	-0.029	-0.065	-0.042	-0.058	-0.038	-0.063	-0.055	-0.066	-0.062	-0.085
Ar-Ar	-0.203	-0.154	-0.210	-0.155	-0.210	-0.187	-0.197	-0.172	-0.200	-0.189	-0.202	-0.192	-0.207

Table S10: Interaction energy components on the TK21/S1 dataset from SAPT(PBE0AC) calculations. The basis set is aug-cc-pVDZ.

PBE0AC	$E^{(1)}_{\mathrm{elst}}$	$E^{(1)}_{\mathrm{exch}}$	$E^{(2)}_{\mathrm{ind}}$	$E^{(2)}_{\mathrm{exch-ind}}$	$E^{(2)}_{\mathrm{disp}}$	$E^{(2)}_{\mathrm{exch-disp}}$	E_{int}
F^--HF	−115.5	121.3	−103.9	47.84	−19.59	6.973	−62.83
F^--H_2O	−60.78	60.96	−44.20	26.07	−12.33	4.557	−25.72
Na^+-H_2O	−39.96	14.37	−24.37	13.81	−0.443	0.105	−36.50
HF-HF	−9.630	8.839	−4.418	2.350	−2.554	0.421	−4.992
H_2O-H_2O	−13.11	1.234	−0.193	3.421	−4.047	0.824	−5.353
NH_3-CH_4	−0.999	13.14	−5.577	0.347	−1.792	0.204	−0.758
NH_3-H_2O	−17.68	1.977	−0.495	5.743	−5.433	1.265	−6.682
C_2H_6-HCN	−1.082	18.45	−9.026	0.358	−1.777	0.146	−1.184
CH_4-CH_4	−0.329	0.522	−0.083	0.187	−1.584	0.142	−0.543
C_2H_2-C_2H_2(PD)	−2.488	2.767	−0.779	0.600	−2.169	0.264	−1.805
C_2H_2-C_2H_2(S)	0.206	0.628	−0.178	0.128	−1.040	0.103	−0.153
C_2H_2-C_2H_2(T)	−2.796	3.109	−0.925	0.572	−2.127	0.255	−1.912
NCCN-NCCN	−3.305	2.091	−0.920	0.982	−3.071	0.364	−2.352
PCCP-PCCP	−2.902	4.014	−1.336	3.225	−7.551	1.100	−2.639
N_2-N_2	−0.231	2.600	−1.304	0.075	−0.664	0.043	−0.338
P_2-P_2	−0.967	0.377	−0.045	1.246	−2.84	0.37	−0.89
N_2O-He(GM)	−0.085	0.183	−0.012	0.029	−0.45	0.02	−0.153
N_2O-He(LM)	−0.033	0.380	−0.046	0.009	−0.24	0.01	−0.083
CO_2-He(GM)	−0.078	0.143	−0.010	0.027	−0.40	0.02	−0.099
CO_2-He(LM)	−0.024	0.267	−0.085	0.006	−0.19	0.01	−0.067
Ar-Ar	−0.079	6.948	−3.459	0.084	−0.41	0.02	−0.208

Results taken from T. Korona, Mol. Phys. 111:3705-3715 (2013)

Table S11: First order electrostatic energy (in milliHartree) on the TK21/S2 dataset from SAPT(CCSD) and SAPT(DFT) calculations. The basis set is aug-cc-pVTZ.

$E^{(1)}_{\mathrm{elst}}$	CCSD	TPSS	TPSS AC	revTPSS	revTPSS AC	MVS	MVS AC	SCAN	SCAN AC	SCAN0	SCAN0 AC	LC-PBETPSS	SAPT2
F^--HF	-114.9	-114.4	-114.4	-114.3	-114.2	-117.2	-117.4	-115.5	-115.6	-117.4	-117.4	-115.9	-115.0
F^--H_2O	-61.31	-60.85	-60.53	-60.79	-60.47	-60.45	-60.57	-60.82	-60.77	-60.89	-60.98	-61.83	-61.76
Na^+-H_2O	-39.44	-39.11	-39.13	-38.83	-38.85	-39.95	-40.04	-39.63	-39.73	-40.45	-40.56	-42.51	-40.00
HF-HF	-9.437	-9.549	-9.396	-9.487	-9.335	-9.460	-9.434	-9.556	-9.508	-9.650	-9.625	-10.07	-9.639
H_2O-H_2O	-13.12	-13.13	-12.88	-13.07	-12.82	-13.07	-13.04	-13.11	-13.03	-13.16	-13.17	-13.75	-13.43
NH_3-CH_4	-0.922	-0.922	-0.914	-0.885	-0.881	-0.882	-0.935	-0.910	-0.943	-0.899	-0.955	-0.96	-0.958
NH_3-H_2O	-17.91	-17.91	-17.59	-17.92	-17.61	-17.93	-17.88	-17.89	-17.74	-17.91	-17.88	-18.62	-18.31
CH_4-CH_4	-0.242	-0.285	-0.296	-0.277	-0.291	-0.197	-0.251	-0.250	-0.291	-0.230	-0.287	-0.242	-0.256
N_2-N_2	-0.251	-0.278	-0.246	-0.276	-0.245	-0.235	-0.230	-0.250	-0.238	-0.224	-0.226	-0.236	-0.265
P_2-P_2	-0.980	-0.970	-0.885	-0.959	-0.880	-0.656	-0.730	-0.883	-0.900	-0.934	-0.957	-1.059	-1.040
N_2O-He(GM)	-0.082	-0.103	-0.078	-0.100	-0.075	-0.079	-0.069	-0.093	-0.076	-0.084	-0.075	-0.077	-0.082
N_2O-He(LM)	-0.034	-0.045	-0.034	-0.043	-0.033	-0.034	-0.031	-0.039	-0.033	-0.035	-0.032	-0.036	-0.034
CO_2-He(GM)	-0.075	-0.097	-0.072	-0.093	-0.071	-0.072	-0.063	-0.086	-0.071	-0.076	-0.068	-0.068	-0.076
CO_2-He(LM)	-0.025	-0.033	-0.025	-0.032	-0.024	-0.025	-0.023	-0.029	-0.025	-0.026	-0.023	-0.027	-0.026
Ar-Ar	-0.086	-0.110	-0.081	-0.109	-0.080	-0.073	-0.069	-0.091	-0.077	-0.083	-0.077	-0.079	-0.089

Table S12: First order exchange energy in the S^2 approximation (in milliHartree) on the TK21/S2 dataset from SAPT(CCSD) and SAPT(DFT) calculations. The basis set is aug-cc-pVTZ.

$E^{(1)}_{\text{exch}}(S^2)$	CCSD	TPSS	TPSS AC	revTPSS	revTPSS AC	MVS	MVS AC	SCAN	SCAN AC	SCAN0	SCAN0 AC	LC-PBETPSS	SAPT2
F^--HF	123.8	128.6	128.3	130.4	130.1	118.4	118.4	124.7	124.3	117.9	117.9	117.2	127.0
F^--H_2O	61.42	64.23	63.73	64.82	64.30	57.99	58.04	62.08	61.90	58.63	58.66	61.67	63.39
Na^+-H_2O	14.36	15.34	14.39	15.28	14.33	13.42	13.25	14.41	14.02	13.91	13.81	15.44	14.28
HF-HF	8.76	10.10	9.181	10.11	9.195	8.315	8.156	9.191	8.776	8.471	8.396	8.541	8.864
H_2O-H_2O	13.08	14.41	13.55	14.42	13.57	12.25	12.23	13.43	13.15	12.63	12.65	13.25	13.34
NH_3-CH_4	1.67	1.941	1.917	1.894	1.885	1.511	1.736	1.752	1.897	1.649	1.885	1.823	1.692
NH_3-H_2O	18.17	19.52	18.88	19.62	19.00	17.29	17.49	18.65	18.56	17.81	17.99	18.79	18.71
CH_4-CH_4	0.926	1.131	1.173	1.090	1.144	0.853	1.054	1.016	1.167	0.949	1.164	1.008	0.933
N_2-N_2	0.501	0.639	0.498	0.629	0.493	0.464	0.437	0.549	0.491	0.505	0.507	0.538	0.484
P_2-P_2	2.536	2.620	2.416	2.590	2.402	1.859	2.076	2.415	2.476	2.538	2.609	2.914	3.154
N_2O-He(GM)	0.379	0.501	0.380	0.485	0.372	0.384	0.341	0.450	0.375	0.406	0.365	0.377	0.367
N_2O-He(LM)	0.180	0.251	0.185	0.241	0.180	0.192	0.169	0.219	0.180	0.192	0.172	0.199	0.178
CO_2-He(GM)	0.379	0.511	0.383	0.495	0.375	0.388	0.344	0.456	0.379	0.403	0.365	0.370	0.373
CO_2-He(LM)	0.138	0.196	0.141	0.189	0.137	0.147	0.129	0.170	0.138	0.147	0.133	0.152	0.133
Ar-Ar	0.278	0.361	0.266	0.358	0.264	0.248	0.233	0.303	0.259	0.278	0.258	0.271	0.285

Table S13: Second order induction energy in the S^2 approximation (in milliHartree) on the TK21/S2 dataset from SAPT(CCSD) and SAPT(DFT) calculations. The basis set is aug-cc-pVTZ.

$E^{(2)}_{\text{ind}}$	CCSD	TPSS	TPSS AC	revTPSS	revTPSS AC	MVS	MVS AC	SCAN	SCAN AC	SCAN0	SCAN0 AC	LC-PBETPSS	SAPT2
F^--HF	-112.4	-118.9	-115.5	-118.9	-115.6	-94.78	-93.82	-104.8	-103.0	-100.3	-99.64	-100.2	-109.8
F^--H_2O	-47.06	-51.81	-49.47	-51.59	-49.29	-41.32	-40.71	-45.93	-44.84	-42.57	-42.34	-43.18	-45.14
Na^+-H_2O	-26.51	-28.19	-26.43	-27.82	-26.13	-22.84	-22.87	-25.22	-24.75	-24.52	-24.62	-27.27	-25.98
HF-HF	-4.669	-5.500	-4.724	-5.462	-4.694	-4.266	-4.089	-4.766	-4.414	-4.430	-4.334	-4.422	-4.548
H_2O-H_2O	-5.929	-6.834	-5.980	-6.776	-5.940	-5.142	-5.029	-5.839	-5.526	-5.470	-5.432	-5.644	-5.734
NH_3-CH_4	-0.418	-0.492	-0.483	-0.476	-0.473	-0.375	-0.443	-0.430	-0.475	-0.403	-0.480	-0.426	-0.404
NH_3-H_2O	-9.243	-10.24	-9.493	-10.17	-9.461	-7.480	-7.740	-8.442	-8.532	-8.221	-8.560	-9.106	-8.994
CH_4-CH_4	-0.120	-0.164	-0.170	-0.157	-0.166	-0.112	-0.151	-0.138	-0.167	-0.122	-0.167	-0.118	-0.111
N_2-N_2	-0.090	-0.131	-0.085	-0.129	-0.085	-0.088	-0.075	-0.104	-0.083	-0.088	-0.085	-0.088	-0.081
P_2-P_2	-1.328	-1.513	-1.226	-1.485	-1.214	-0.852	-0.979	-1.240	-1.223	-1.287	-1.302	-1.464	-1.541
N_2O-He(GM)	-0.049	-0.074	-0.045	-0.070	-0.044	-0.038	-0.041	-0.053	-0.045	-0.052	-0.046	-0.050	-0.045
N_2O-He(LM)	-0.012	-0.021	-0.012	-0.020	-0.011	-0.009	-0.010	-0.014	-0.011	-0.013	-0.012	-0.014	-0.010
CO_2-He(GM)	-0.047	-0.070	-0.045	-0.067	-0.044	-0.039	-0.041	-0.053	-0.046	-0.052	-0.047	-0.050	-0.045
CO_2-He(LM)	-0.010	-0.016	-0.010	-0.015	-0.010	-0.009	-0.009	-0.011	-0.010	-0.011	-0.010	-0.011	-0.009
Ar-Ar	-0.100	-0.160	-0.090	-0.159	-0.090	-0.096	-0.078	-0.124	-0.086	-0.105	-0.087	-0.089	-0.098

Table S14: Second order exchange-induction energy in the S^2 approximation (in milliHartree) on the TK21/S2 dataset from SAPT(CCSD) and SAPT(DFT). The basis set is aug-cc-pVTZ.

$E^{(2)}_{\mathrm{exch-ind}}(S^2)$	CCSD	TPSS	TPSS AC	revTPSS	revTPSS AC	MVS	MVS AC	SCAN	SCAN AC	SCAN0	SCAN0 AC	LC-PBETPSS	SAPT2
F^--HF	53.55	61.91	59.18	62.16	59.44	42.87	42.10	50.76	49.27	45.38	44.81	44.31	42.63
F^--H_2O	27.78	33.95	31.73	33.82	31.62	24.58	23.95	28.54	27.48	24.73	24.47	25.28	22.58
Na^+-H_2O	14.96	16.70	15.24	16.42	15.02	12.74	12.82	14.41	14.06	13.81	13.93	15.79	14.34
HF-HF	2.499	3.392	2.624	3.380	2.619	2.361	2.179	2.757	2.400	2.397	2.302	2.252	2.133
H_2O-H_2O	3.644	4.713	3.835	4.679	3.819	3.209	3.091	3.794	3.471	3.379	3.334	3.397	3.217
NH_3-CH_4	0.267	0.355	0.344	0.343	0.338	0.239	0.302	0.293	0.334	0.261	0.334	0.270	0.239
NH_3-H_2O	5.712	6.972	6.255	6.920	6.237	4.399	4.678	5.334	5.431	5.013	5.357	5.611	5.231
CH_4-CH_4	0.111	0.160	0.167	0.153	0.163	0.106	0.147	0.133	0.163	0.116	0.162	0.111	0.104
N_2-N_2	0.080	0.122	0.076	0.120	0.075	0.081	0.067	0.096	0.074	0.081	0.078	0.079	0.073
P_2-P_2	1.249	1.460	1.176	1.433	1.164	0.818	0.939	1.192	1.171	1.234	1.246	1.404	1.472
N_2O-He(GM)	0.032	0.056	0.029	0.053	0.028	0.025	0.026	0.037	0.029	0.035	0.029	0.030	0.026
N_2O-He(LM)	0.010	0.019	0.009	0.018	0.009	0.008	0.009	0.011	0.009	0.010	0.009	0.009	0.005
CO_2-He(GM)	0.028	0.049	0.026	0.047	0.025	0.023	0.024	0.033	0.026	0.030	0.026	0.026	0.021
CO_2-He(LM)	0.006	0.012	0.006	0.011	0.006	0.005	0.005	0.007	0.006	0.006	0.006	0.006	0.004
Ar-Ar	0.098	0.159	0.088	0.158	0.088	0.095	0.076	0.122	0.084	0.104	0.085	0.087	0.096

Table S15: Second order dispersion energy in the S^2 approximation (in milliHartree) on the TK21/S2 dataset from SAPT(CCSD) and SAPT(DFT). The basis set is aug-cc-pVTZ.

$E^{(2)}_{\rm disp}$	CCSD	TPSS	TPSS AC	revTPSS	revTPSS AC	MVS	MVS AC	SCAN	SCAN AC	SCAN0	SCAN0 AC	LC-PBETPSS	SAPT2 +(CCD)
F^--HF	-25.38	-25.07	-24.55	-25.11	-24.60	-21.60	-21.39	-23.25	-22.92	-22.39	-22.27	-21.32	-25.09
F^--H_2O	-15.58	-15.88	-15.38	-15.86	-15.38	-13.50	-13.34	-14.68	-14.42	-14.01	-13.94	-13.91	-15.21
Na^+-H_2O	-0.876	-0.949	-0.867	-0.951	-0.868	-0.806	-0.778	-0.854	-0.813	-0.828	-0.808	-0.887	-0.848
HF-HF	-3.179	-3.305	-3.160	-3.301	-3.158	-2.775	-2.750	-3.016	-2.953	-2.899	-2.887	-2.867	-3.111
H_2O-H_2O	-4.902	-4.993	-4.821	-4.977	-4.810	-4.222	-4.207	-4.598	-4.538	-4.478	-4.475	-4.520	-4.796
NH_3-CH_4	-1.978	-1.981	-1.975	-1.950	-1.948	-1.716	-1.781	-1.871	-1.914	-1.855	-1.920	-1.904	-1.920
NH_3-H_2O	-6.481	-6.561	-6.412	-6.544	-6.401	-5.553	-5.587	-6.031	-6.013	-5.936	-5.977	-6.116	-6.358
CH_4-CH_4	-1.666	-1.670	-1.686	-1.636	-1.656	-1.473	-1.546	-1.601	-1.656	-1.594	-1.667	-1.622	-1.617
N_2-N_2	-0.753	-0.767	-0.733	-0.761	-0.729	-0.655	-0.655	-0.713	-0.705	-0.709	-0.715	-0.735	-0.736
P_2-P_2	-3.516	-3.288	-3.232	-3.267	-3.216	-2.717	-2.804	-3.133	-3.169	-3.265	-3.300	-3.434	-3.676
N_2O-He(GM)	-0.552	-0.563	-0.526	-0.553	-0.519	-0.476	-0.475	-0.532	-0.517	-0.526	-0.517	-0.545	-0.541
N_2O-He(LM)	-0.290	-0.294	-0.275	-0.289	-0.271	-0.250	-0.249	-0.276	-0.268	-0.272	-0.268	-0.291	-0.288
CO_2-He(GM)	-0.494	-0.506	-0.472	-0.497	-0.465	-0.424	-0.423	-0.476	-0.462	-0.465	-0.457	-0.481	-0.483
CO_2-He(LM)	-0.227	-0.233	-0.218	-0.229	-0.215	-0.197	-0.196	-0.218	-0.212	-0.214	-0.211	-0.228	-0.223
Ar-Ar	-0.554	-0.564	-0.538	-0.562	-0.536	-0.475	-0.475	-0.522	-0.514	-0.518	-0.515	-0.520	-0.550

Table S16: Second order exchange-dispersion energy in the S^2 approximation (in milliHartree) on the TK21/S2 dataset from SAPT(CCSD) and SAPT(DFT). The basis set is aug-cc-pVTZ.

$E^{(2)}_{\mathrm{exch-disp}}(S^2)$	CCSD	TPSS	TPSS AC	revTPSS	revTPSS AC	MVS	MVS AC	SCAN	SCAN AC	SCAN0	SCAN0 AC	LC-PBETPSS	SAPT2
F^--HF	8.377	8.816	8.393	8.818	8.401	7.123	6.977	7.908	7.669	7.343	7.245	6.820	6.313
F^--H_2O	5.367	5.832	5.507	5.815	5.496	4.657	4.559	5.207	5.043	4.786	4.743	4.686	3.942
Na^+-H_2O	0.164	0.191	0.168	0.190	0.167	0.153	0.146	0.166	0.156	0.158	0.154	0.176	0.135
HF-HF	0.527	0.613	0.540	0.612	0.539	0.464	0.449	0.527	0.493	0.481	0.473	0.460	0.380
H_2O-H_2O	1.003	1.101	1.004	1.096	1.001	0.846	0.833	0.958	0.921	0.900	0.895	0.905	0.767
NH_3-CH_4	0.212	0.233	0.231	0.227	0.226	0.178	0.199	0.207	0.221	0.198	0.221	0.209	0.159
NH_3-H_2O	1.505	1.602	1.518	1.595	1.515	1.207	1.228	1.382	1.373	1.328	1.353	1.386	1.204
CH_4-CH_4	0.133	0.149	0.154	0.143	0.149	0.114	0.134	0.134	0.150	0.128	0.149	0.130	0.097
N_2-N_2	0.049	0.059	0.049	0.058	0.048	0.042	0.040	0.050	0.046	0.047	0.047	0.049	0.041
P_2-P_2	0.440	0.437	0.409	0.432	0.406	0.297	0.324	0.392	0.400	0.418	0.427	0.459	0.548
N_2O-He(GM)	0.025	0.032	0.024	0.031	0.024	0.021	0.021	0.027	0.024	0.025	0.023	0.024	0.019
N_2O-He(LM)	0.011	0.015	0.011	0.014	0.011	0.010	0.010	0.012	0.011	0.011	0.010	0.012	0.008
CO_2-He(GM)	0.023	0.029	0.022	0.028	0.022	0.019	0.019	0.024	0.021	0.022	0.021	0.021	0.016
CO_2-He(LM)	0.008	0.011	0.008	0.010	0.008	0.007	0.007	0.009	0.008	0.008	0.008	0.009	0.005
Ar-Ar	0.028	0.035	0.027	0.035	0.027	0.023	0.022	0.029	0.025	0.027	0.025	0.025	0.023

Table S17: Interaction energies on the TK21/S2 dataset from SAPT(CCSD) and SAPT(DFT) calculations. The basis set is aug-cc-pVTZ.

$E_{\rm int}^{\rm SAPT}$	CCSD	TPSS	TPSS AC	revTPSS	revTPSS AC	MVS	MVS AC	SCAN	SCAN AC	SCAN0	SCAN0 AC	LC-PBETPSS	SAPT2 +(CCD)
F^--HF	-66.94	-59.02	-58.59	-56.93	-56.51	-65.20	-65.21	-60.27	-60.33	-69.48	-69.36	-69.13	-73.98
F^--H_2O	-29.39	-24.52	-24.43	-23.80	-23.73	-28.04	-28.06	-25.60	-25.60	-29.33	-29.38	-27.27	-32.20
Na^+-H_2O	-37.34	-36.01	-36.62	-35.72	-36.33	-37.30	-37.48	-36.72	-37.06	-37.92	-38.09	-39.26	-38.07
HF-HF	-5.499	-4.245	-4.935	-4.151	-4.835	-5.362	-5.491	-4.862	-5.206	-5.629	-5.676	-6.103	-5.922
H_2O-H_2O	-6.223	-4.737	-5.300	-4.628	-5.178	-6.130	-6.122	-5.364	-5.546	-6.201	-6.204	-6.359	-6.638
NH_3-CH_4	-1.166	-0.866	-0.880	-0.848	-0.853	-1.045	-0.922	-0.959	-0.880	-1.049	-0.916	-0.984	-1.192
NH_3-H_2O	-8.247	-6.613	-6.839	-6.500	-6.715	-7.852	-7.804	-6.990	-6.922	-7.916	-7.720	-8.052	-8.523
CH_4-CH_4	-0.858	-0.678	-0.659	-0.683	-0.658	-0.709	-0.614	-0.705	-0.635	-0.753	-0.645	-0.733	-0.851
N_2-N_2	-0.464	-0.355	-0.442	-0.358	-0.442	-0.392	-0.416	-0.372	-0.415	-0.389	-0.395	-0.392	-0.484
P_2-P_2	-1.599	-1.253	-1.343	-1.256	-1.339	-1.246	-1.173	-1.257	-1.245	-1.296	-1.276	-1.180	-1.082
N_2O-He(GM)	-0.247	-0.152	-0.216	-0.155	-0.214	-0.164	-0.197	-0.164	-0.210	-0.196	-0.220	-0.240	-0.255
N_2O-He(LM)	-0.135	-0.075	-0.115	-0.079	-0.116	-0.084	-0.103	-0.087	-0.113	-0.107	-0.120	-0.120	-0.139
CO_2-He(GM)	-0.186	-0.084	-0.158	-0.087	-0.157	-0.106	-0.140	-0.102	-0.152	-0.138	-0.161	-0.182	-0.195
CO_2-He(LM)	-0.110	-0.063	-0.098	-0.066	-0.098	-0.071	-0.087	-0.072	-0.095	-0.088	-0.098	-0.099	-0.115
Ar-Ar	-0.336	-0.280	-0.328	-0.280	-0.327	-0.278	-0.290	-0.283	-0.309	-0.298	-0.310	-0.305	-0.334

Table S18: Interaction energy components on the TK21/S2 dataset from SAPT(PBE0AC) calculations. The basis set is aug-cc-pVTZ.

PBE0AC	$E^{(1)}_{\mathrm{elst}}$	$E^{(1)}_{\mathrm{exch}}$	$E^{(2)}_{\mathrm{ind}}$	$E^{(2)}_{\mathrm{exch-ind}}$	$E^{(2)}_{\mathrm{disp}}$	$E^{(2)}_{\mathrm{exch-disp}}$	E_{int}
F^--HF	−116.2	122.0	−109.96	52.844	−23.72	7.912	−67.13
F^--H_2O	−60.93	60.98	−46.286	27.947	−14.83	5.154	−27.96
Na^+-H_2O	−40.12	14.48	−26.305	15.152	−0.870	0.169	−37.49
HF-HF	−9.574	8.828	−4.633	2.497	−3.078	0.513	−5.447
H_2O-H_2O	−13.10	13.14	−5.851	3.646	−4.741	0.967	−5.937
NH_3-CH_4	−0.986	1.976	−0.506	0.355	−2.018	0.238	−0.941
NH_3-H_2O	−17.79	18.51	−9.396	6.051	−6.344	1.476	−7.497
CH_4-CH_4	−0.301	1.223	−0.176	0.172	−1.744	0.161	−0.666
N_2-N_2	−0.234	0.525	−0.089	0.081	−0.747	0.050	−0.414
P_2-P_2	−0.961	2.608	−1.331	1.274	−3.386	0.443	−1.353
N_2O-He(GM)	−0.076	0.374	−0.047	0.030	−0.541	0.025	−0.235
N_2O-He(LM)	−0.035	0.181	−0.012	0.009	−0.282	0.011	−0.128
CO_2-He(GM)	−0.070	0.382	−0.048	0.027	−0.481	0.022	−0.168
CO_2-He(LM)	−0.026	0.142	−0.010	0.006	−0.223	0.008	−0.103
Ar-Ar	−0.081	0.269	−0.091	0.089	−0.538	0.027	−0.325

Results taken from T. Korona, Mol. Phys. 111:3705-3715 (2013)

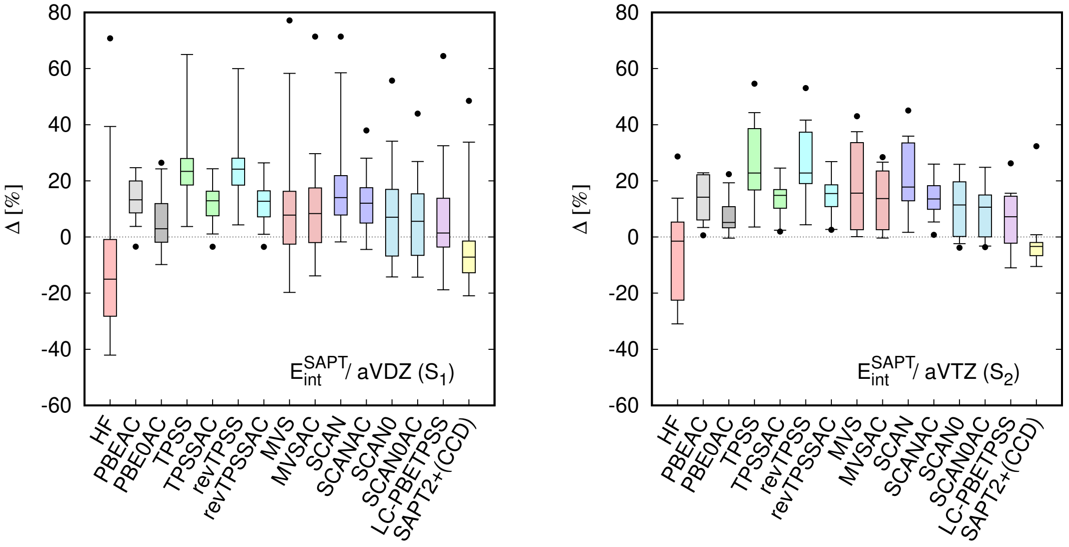

Figure S5: Box plot of relative percent errors of SAPT interaction energies for dimers of the TK21 data set calculated in aug-cc-pVDZ (left) and aug-cc-pVTZ (right) basis sets. Errors with respect to SAPT(CCSD) results.
